# Exploring the prebiotic potential of commercial cellobiose: a randomized, controlled trial

**DOI:** 10.1017/gmb.2026.10032

**Published:** 2026-07-27

**Authors:** Manxi Huang, Peter Philip James Jackson, Afroditi Chatzifragkou, Robert Adrian Rastall

**Affiliations:** 1 Food and Nutritional Sciences, University of Reading, UK; 2 Department of Food Science, Purdue University, USA

**Keywords:** cellobiose, prebiotic, gut microbiome, quantitative microbiome profiling, dietary intervention trial

## Abstract

Cellobiose, a β-(1 → 4)-linked disaccharide indigestible by humans, is a novel candidate prebiotic. Evidence from controlled human trials on its effects on the gut microbiota and metabolites remains limited. We conducted an exploratory randomised, double-blind, placebo-controlled trial in 37 healthy adults. Participants were allocated to three arms for 4 weeks, received 5 g/day and 10 g/day for cellobiose, oligofructose P95, or maltodextrin placebo. The primary endpoint was change in absolute abundance of *Bifidobacterium* and *Lactobacillus* (measured by 16S rRNA gene sequencing). Secondary endpoints included other taxa, diversity, faecal short-chain fatty acids (SCFAs) measured by gas chromatography–mass spectrometry and gastrointestinal (GI) tolerability. Cellobiose did not significantly change *Bifidobacterium* or *Lactobacillus* versus baseline or placebo (*P* > 0.05). Oligofructose P95 induced a borderline increase in *Bifidobacterium* (*P* = 0.049). Overall community composition remained unchanged. However, network analyses under cellobiose revealed tighter positive correlations among key genera. Faecal SCFA levels were not altered. GI symptom rates were low and similar across all arms. Cellobiose, at doses up to 10 g/day, is safe and well tolerated but did not enrich classic beneficial taxa. The findings suggest cellobiose drives subtle community shifts towards butyrate-producing bacteria.

## Introduction

Modulation of the gut microbiota is an approach to support human health (Schmidt et al., 2018; Valdes et al., 2018; Hou et al., 2022; Ugwu et al., 2024). Some bacterial species are significantly associated with various health effects (Zafar and Saier, 2021; Negishi et al., 2025). *Bifidobacterium* and *Lactobacillus* have served as key genera due to their barrier-protection effects against inflammatory diseases and association with enhanced host immune system (O’Callaghan and van Sinderen, 2016; Dempsey and Corr, 2022). Prebiotics are substrates, selectively utilized by host microorganisms, conferring health benefits (Gibson et al., 2017). Most established prebiotics (e.g., inulin-type fructans, galacto-oligosaccharides) only target a narrow group of taxa (primarily bifidobacteria). Novel prebiotic candidates are needed to expand the range of selectively stimulated microbial communities beyond *Bifidobacterium.*

Cellobiose, a β-(1 → 4) linked glucose disaccharide, is potentially a novel prebiotic. It can be either derived enzymatically from cellulose in plant-based biomass or synthesized enzymatically from glucose and sucrose. Recent EU regulatory approval for its use in foods (Regulation 2023/943), based on a safety assessment submitted by Turck et al. (2022), allows human consumption of cellobiose, enabling dietary intervention studies. Although safety is established, the specific impact on the human gut microbiota at a community-wide level remains unknown. This study addresses this gap. To our knowledge, this is the first randomised control human trial to apply high throughput 16S rRNA gene sequencing to investigate how cellobiose modulates gut microbiota structure and metabolic outputs. The aim of the study was to move beyond the analysis of targeted bacterial groups by profiling the full bacterial community, allowing detection of broad shifts in diversity and specific compositional changes.

## Methods

### Ethics

The study was given ethical approval by the University of Reading Research Ethics Committee [UREC (24/20)] and was conducted in accordance with the Declaration of Helsinki. The study was registered at clinicaltrials.gov (NCT07097389). All participants gave written consent prior to study entry.

### Volunteer recruitment and screening

Healthy adults (18 years old and above) were recruited in Reading, UK. Inclusion required no history of gastrointestinal (GI) disorders or food allergies, and regular bowel habits (≥3 movements/week). Exclusion criteria were antibiotic use within last 6 months; anaemia or any chronic/acute illness (e.g., pre-diabetes); cancer diagnosis or history of bowel resection; active smoking, alcohol/drug misuse; pregnancy or lactation.

### Study design and interventions

This randomized, double-blind, parallel-group, placebo-controlled trial involved a 4-week intervention: 5 g/day (Week 0–1; adaptation) increasing to 10 g/day (Weeks 2–4). We chose a parallel design instead of a cross-over design to avoid unpredictable carry-over effects, ensuring that each group’s baseline remained completely independent.

Prior to enrolment, eligible participants received both verbal and written information about the study and provided informed consent. Participants maintained their habitual diet but avoided other prebiotics/probiotics. The study interventions included: commercial cellobiose (Savanna GmbH, Germany), oligofructose (Orafti® P95, DP 3–9, average DP 4; Beneo-Orafti, Tienen, Belgium), and a maltodextrin placebo (Special Ingredients Ltd, UK). Participants were randomized (1:1:1) using a computer-generated sequence (https://www.randomizer.org/), and allocated via an online tool (https://ctrandomization.cancer.gov/). Blinding was ensured by identical packaging labelled with random letter codes; the decoding key was withheld from all personnel until analysis. All intervention bottles looked identical and bore only the letter code.

Stool samples were collected at baseline (V1) and post-intervention (V5). Compliance (≥95%) was verified via returned bottles and daily diaries. Each entry recorded the date and time of product consumption and daily tick-boxes for GI events (bloating; nausea; gas/flatulence; vomiting; abdominal pain or cramps; changes in stool consistency; diarrhoea; constipation; and frequency of bowel movements). All data were captured and managed using the secure Research Electronic Data Capture system hosted at the University of Reading, which facilitates validated data entry, audit trails, automated exports, and the integration of external data.

### Outcome measures

Primary outcomes: Change in absolute abundance of faecal *Bifidobacterium* spp. and *Lactobacillus* spp. (16S rRNA sequencing and FISH-FLOW), changes in total faecal short-chain fatty acid (SCFA) concentration from V1 to V5.

Secondary outcomes: Magnitude and pattern of overall gut-microbiota shift from V1 to V5 and GI tolerance and safety (daily symptom checkboxes; adverse events) during Days 1–28. Exploratory outcomes: SCFA-microbe correlations for each subject, log₂-fold-changes (V5 vs. V1) in stool acetate, propionate, and butyrate. Spearman correlation coefficients (*ρ*) with 95% confidence intervals (CIs) were calculated to assess the relationship between the change in each SCFA and the log₂-fold-changes in *Bifidobacterium.* For other key genera (e.g., *Faecalibacterium*, *Blautia*, *Roseburia*, and *Bacteroides*), compositional changes were reported as mean log₂-fold-changes with 95% CIs. As an exploratory analysis, no formal hypothesis tests were performed; all results are presented as effect-size estimates only.

### Faecal sample collection and processing

Participants received sterile stool collection containers for sample collection on V1 and V5. Immediately after collection, samples were placed in insulated bags with ice packs and retrieved from the participants’ residences within 2 hours of voiding. Additionally, a 5 g portion of each sample was diluted at a ratio of 1:10 (w/w) in anaerobic phosphate-buffered saline (PBS; 0.1 M, pH 7.4) and homogenized using a stomacher at 230 paddle beats per minute for 2 minutes at room temperature. Following homogenization, 750 μL aliquots of the faecal slurry were centrifuged at 13,000 g for 5 minutes. Supernatants were removed, and pellets resuspended in 375 μL of PBS (0.1 M, pH 7.4) before fixing in 1125 μL of 4% (w/v) paraformaldehyde for 4 hours at 4 °C. After fixing, the samples were centrifuged again at 13,000 g for 5 minutes at room temperature, washed with 1 mL of PBS, and washing was repeated twice. The final pellet was resuspended in 300 μL of PBS and stored in a 1:1 (v/v) PBS–ethanol solution at −20 °C until further analysis by FISH. In parallel, 1.5-mL aliquots of the faecal slurry were centrifuged at 13,000 g for 10 minutes, and the supernatants were transferred to 1.5-mL tubes and stored at −80 °C for subsequent analyses. The pellets were saved for bacterial DNA extraction at −80 °C.

### *Enumeration of faecal microbial populations by* FISH-FLOW

FISH-FLOW analysis was performed following Jackson et al. (2023). The probes used are detailed in Table 1. Fluorescence measurements were taken at 488 and 640 nm using a BD Accuri C6 Plus flow cytometer. To minimize background noise, thresholds were set at 9000 for the forward scatter and 3000 for the side scatter, with a gate established to capture 90% of the primary event population. The flow rate was maintained at 10 μL/min, and data were acquired until 100,000 events were collected, as managed by the Accuri CFlow Sampler software. Bacterial counts were calculated by adjusting flow cytometry data according to PBS dilution, with results expressed as the logarithm (log₁₀) of cells per gram of fresh, wet faeces.

**Table 1.** Name, sequence, and target group of oligonucleotide probes used in bacterial enumeration. Table 1. long description.

Probes	Sequence (5′ to 3′)	Targeted groups	Reference
Non Eub	ACTCCTACGGGAGGCAGC	Control probe complementary to EUB338; non bacteria	Wallner et al. (1993)
Eub 338 I	GCTGCCTCCCGTAGGAGT	Most bacteria	Amann et al. (1990)
Eub 338 I-II	GCAGCCACCCGTAGGTGT	Most bacteria	Daims et al. (1999)
Eub 338 I-II-III	GCTGCCACCCGTAGGTGT	Most bacteria	Daims et al. (1999)

### Bacterial DNA extraction

Fresh stool (1.5 g) was stored at −80 C for metabolic profiling analysis conducted after the intervention study was completed. Bacterial DNA was extracted from faecal samples using QIAamp PowerFaecal (QIAGEN) according to the manufacturer’s instructions. In summary, faecal samples were homogenized and aliquoted into 2 mL screwcap tubes containing 0.6 g 0.1 mm glass beads. Bead beating was performed using a Fastprep24 (MP Biomedicals) for 4 cycles of 45 s at speed 5. Then, 500 μL of raw extract were then used for DNA isolation.

### DNA isolation, library preparation, and 16S rRNA gene sequencing

Extracted bacterial DNA was amplified by polymerase chain reaction (PCR) of the V4 region of the 16S rRNA gene with two-stage Nextera PCR libraries using the primer pairs 515F (50-GTG YCA GCM GCC GCG GTA A-30) and 806R (50-GGA CTA CNV GGG TWT CTA AT-30).

Raw sample extracts were diluted to 2.5 ng/μL using Tris buffer, and 5 μL were used in first step PCR, together with 5x HOT FIREPol MultiPlex Mix (Solis BioDyne) and 4 μM primer mix (forward and reverse) 515F/806R (Microsynth). First step PCR samples were purified with NGS Clean Beads (Labgene). Bead ratio was 1:1:2, and beads were washed with 75% ethanol, air-dried, and resuspended in the Tris buffer. In second-step PCR, each sample was individually barcoded, using Nextera XT Index Kit v2 (Illumina, San Diego, California) and 5x HOT FIREPol MultiPlex Mix (Solis BioDyne). Second-step PCR samples were purified with NGS Clean Beads (Labgene). The final second-step PCR products were quantified using a Quant-iT PicoGreen dsDNA Assay Kit (Thermo Fisher Scientific). Amplicons were pooled in equimolar amounts prior to sequencing. The final pool was quantified using a Quant-iT Pico-Green dsDNA Assay Kit (Thermo Fisher Scientific) and Fragment analyser (Agilent).

Subsequent PCR libraries were sequenced on an Illumina MiSeq platform using a v2 500 (2250 bp read length). Pools were diluted to 9.2 pM and loaded together with 15% PhiX (Illumina, FC-110-3001) to increase diversity resulting in a raw cluster density of 631 and a cluster passed filter rate of 98%. Paired-end reads that passed Illumina’s chastity filter were subjected to demultiplexing and trimming of Illumina adaptor residuals using Illumina’s bcl2fastq software version v2.20.0.422. The quality of the reads was checked with the software FastQC version 0.11.8, and sequencing reads that fell below an average Q-score of 20 or had any uncalled bases (N) were removed from further analysis. The locus-specific V4 primers were trimmed from the sequencing reads with the software cutadapt v3.2. Paired-end reads were discarded if the primer could not be trimmed. Trimmed forward and reverse reads of each paired-end read were merged to reform *in silico* the sequenced molecule considering a minimum overlap of 15 bases using the software USEARCH version 11.0.667. Merged sequences were again quality filtered, allowing a maximum of one expected erroneous base per merged read. Reads that contain ambiguous bases or were outliers regarding the amplicon size distribution were also discarded. Samples that resulted in less than 5000 merged reads were discarded so as not to distort the statistical analysis. Remaining reads were denoised using the UNOISE algorithm implemented in USEARCH to form amplicon sequencing variants (ASVs), discarding singletons and chimeras in the process. The resulting ASV abundance table was then filtered for possible barcode bleed-in contaminations using the UNCROSS algorithm. ASV sequences were compared to the reference sequences of the RDP 16S database provided by https://www.drive5.com/usearch/manual/sintax_downloads.html, and taxonomies were predicted considering a minimum confidence threshold of 0.5 using the SINTAX algorithm implemented in USEARCH. The resulting library was then corrected by taking into consideration numbers of 16S copies and rarefying to an even sampling intensity to reduce bias in diversity metric calculations and quantified as described by Vandeputte et al. (2017).

### Organic acids by gas chromatography-flame ionization detection

Faecal organic acids were extracted and quantified by gas chromatography-flame ionization detection (GC-FID) according to a modified Richardson et al. (1989) protocol. Briefly, the previously collected faecal fluids (1.5 mL) were thawed, and centrifuged at 11,337 × g for 10 min. One millilitre of sample was transferred into a labelled 100 mm × 16 mm glass tube (International Scientific Supplies Ltd, Bradford, UK) and 50 μL of 2-ethylbutyric acid (0.1 mol/L, internal standard), 500 μL concentrated HCl and 3 ml diethyl ether were added to each glass tube before vortexing for 1 min. Samples were centrifuged at 2,000 × g for 10 min. The resulting diethyl ether (upper) layer of each sample was transferred to clean 100 mL screw top glass tubes. Ether extract (400 μL) and 50 μL *N*-tert-butyldimethylsilyl)-*N*-methyl trifluoroacetamide were added into a GC screw-cap vial. Samples were left at room temperature for 72 h to allow samples to completely derivatise. An Agilent/HP 6890 Gas Chromatograph (Hewlett Packard, UK) using an HP-5MS 30 m × 0.25 mm column with a 0.25 μm coating (cross-linked [5%-phenyl]-methylpolysiloxane, Hewlett Packard) was used for analysis of organic acids. Temperatures of injector and detector were 275 °C , with the column temperature programmed from 63 °C to 190 °C at 15 °C /min followed by 190 °C for 3 minutes. Helium was the carrier gas (flow rate 1.7 ml/min; head pressure 133 KPa). A split ratio of 100:1 was used. Calibration curves were generated using acetic, propionic, butyric, lactate, iso-butyric, iso-valeric, valeric, and succinic acids (12.5–100 mmol/L). Raw concentrations (mmol/L) were converted to total SCFA per gram of starting material by multiplying by the extract volume and dividing by the wet weight of faeces. Final SCFA levels are reported as μmol SCFA per g wet faeces (mean ± standard error [SE]).

### Sample size and statistical analysis

Data were processed and analysed in RStudio 4.2 and Statistical Package for Social Science version 29.0.2.0 (SPSS Inc.). All graphs were generated in GraphPad Prism Version 10.00 for MacOS (GraphPad Software). To investigate the primary, secondary and exploratory outcomes, different statistical analysis were applied. Power analysis (80%, α =0.05) indicated 11 subjects per arm to detect a 20% change in *Lactobacillus* abundance. Twelve subjects per arm (*n* = 36) were planned to be enrolled to account for 10% dropout. In practice, 37 participants were randomised. All randomised participants were included in the intention-to-treat analyses; models are robust to unequal group sizes.

Assumption checks were first applied to check the normality of each outcome (FISH-FLOW, quantitative microbiome profiling [QMP] and metabolic outputs) and of model residuals was assessed by Kolmogorov–Smirnov and Shapiro–Wilk tests, together with Q-Q plots, homogeneity of variances was evaluated by Levene’s test (based on means, medians, and trimmed means). Linearity, homoscedasticity, and homogeneity of regression slopes were examined in the context of the general linear model (GLM; scatterplots of covariate vs. dependent variable, residuals vs. predicted values, and tests of covariate × factor interactions).

For primary and secondary outcomes that met normality and variance-equality assumptions, repeated-measures GLMs (time as within-participant factor; treatment as between-participant factor) were applied to test change over time and treatment × time interactions in bacteriology outcomes. To adjust for baseline differences and participant sex, factorial analysis of covariance (ANCOVA) was performed on change-from-baseline values with baseline and sex as covariates. Pair-wise comparisons within each ANOCVA, repeated-measures general linear analysis of variance were Bonferroni-adjusted for type Ι errors. Outcomes failing normality of residuals (even after log- or other transformations) were analysed non-parametrically. Wilcoxon signed-rank tests were applied for within-intervention changes, and the Kruskal–Wallis H tests were also used for between-intervention comparisons, followed by Dunn’s post-hoc tests with Benjamini–Hochberg false discovery rate (BH-FDR) adjustment. For exploratory outcomes, correlations between bacterial taxa and SCFAs were analysed using log [fold change (post-pre)] using non-parametric Spearman’s rank correlation corrected for using FDR. Tests were two-tailed and *P* value <= 0.05 were considered statistically significant.

## Results

### Participant flow and baseline characteristic

**Figure 1.**
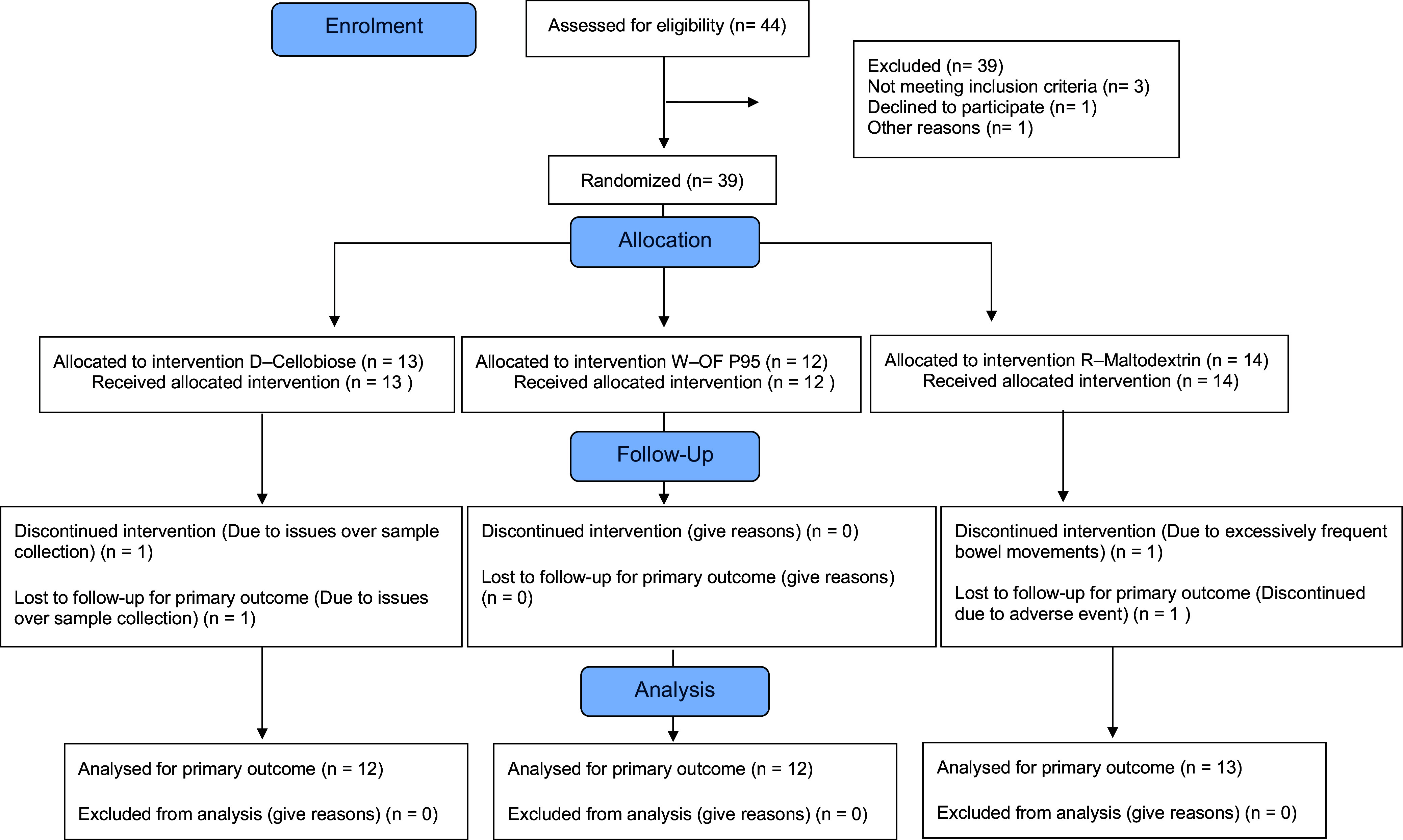
CONSORT flow diagram of participant progress through the phases of a three group, parallel randomised trial (enrolment, intervention allocation, follow-up, and data analysis). Abbreviations: OF P95, oligofructose; CONSORT, Consolidated Standards of Reporting Trials. Figure 1. long description.

In total, there were 44 volunteers who expressed interest and were screened for eligibility, of whom 39 were randomised (*n* = 13 in each group). Two volunteers withdrew from the trial, 37 volunteers completed it (14 males and 23 females) (*n* = 12, 12, 13 for each group) and were assessed in analysis for all primary, secondary, and exploratory outcomes. The Consolidated Standards of Reporting Trials (CONSORT) flow diagram is presented in Figure 1 in accordance with the CONSORT 2025 statement (Hopewell et al., 2025). Participants were recruited for the study between August 2024 and March 2025. The follow-up period for all participants concluded in April 2025, and the trial was completed as planned.

Based on returned bottle counts and consumption diaries, the mean adherence rate was 98% in the cellobiose group, 97% in the maltodextrin group and 99% in the OF P95 group. All participants included in the final analysis met the pre-defined compliance threshold of ≥95%. Table 2 summarizes baseline subject characteristics.

**Table 2.** Baseline participant characteristics Table 2. long description.

Characteristic	Cellobiose(*n* = 12)	OF P95(*n* = 12)	Maltodextrin(*n* = 13)	Total(*n* = 37)
Age (y)	33.8 ± 17.2 (22–72)	37.1 ± 15.2 (20–82)	34.1 ± 12.0 (20–59)	35.0 ± 14.6 (20–82)
Male, n (%)	3 (25%)	6 (50%)	5 (38.5%)	14 (37.8%)
Female, n (%)	9 (75%)	6 (50%)	8 (61.5%)	23 (62.2%)

*Note:* Data are presented as standard error (SE) (range) for age, and n (%) for sex distribution. Abbreviation: OF P95, oligofructose P95.

### GI tolerability and bowel habit

For each GI symptom (bloating, nausea, gas/flatulence, vomiting, abdominal pain or cramps, diarrhoea, constipation), stool-frequency change, and stool-consistency change, we calculated the proportion of days (0–1) that was reported per participant (weekly counts ÷ 7). Median weekly proportions did not differ significantly across cellobiose, OF P95, and maltodextrin arms for any GI symptom (all Kruskal–Wallis qFDR >0.05). Gas/flatulence was the most reported GI symptom, occurring on a mean of 16.6% of study days across all participants (vs. 13.1% for stool-frequency changes and 10.4% for bloating). One participant in the maltodextrin arm withdrew early due to excessively frequent bowel movements that conflicted with their work. No other adverse events were reported. In short, these results indicate comparable GI tolerability among cellobiose, oligofructose, and maltodextrin at the tested doses, with low and stable rates of symptoms and stool changes throughout the 4-week intervention.

### Bacterial enumeration by FISH-FLOW

**Figure 2.**
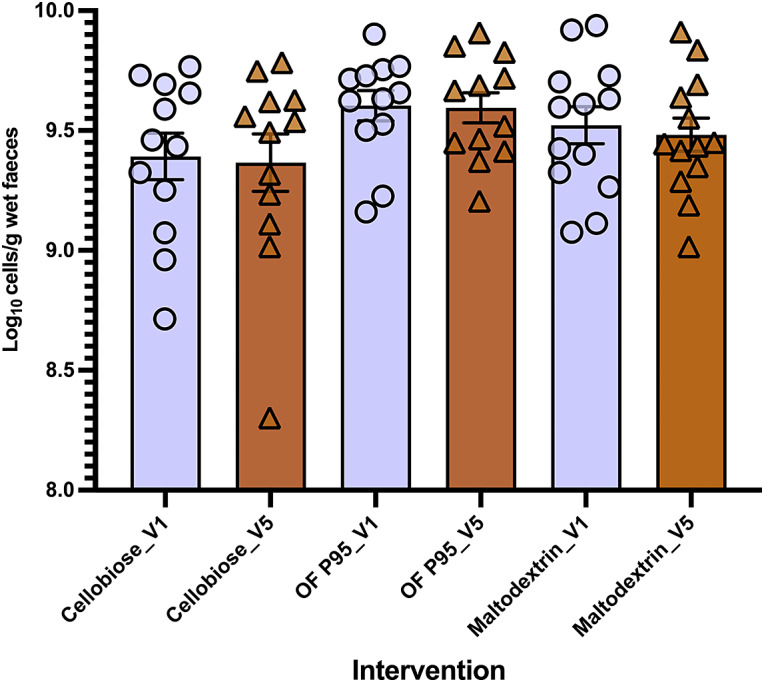
Differences in total bacteria measured by FISH-FLOW (log10 cells/g wet faeces) at visit 5 of the intervention using the Eub I-II-III probes. Values are mean and standard error (SE) (all points) expressed as log10 cells/g wet faeces. Results that are statistically significant between interventions are displayed by specified P values. Figure 2. long description.

All 37 participants provided paired stool samples at baseline (Visit 1) and after 4 weeks (Visit 5). Total bacterial counts, as measured by FISH-FLOW, did not change significantly from baseline to the end of the intervention in any of the three groups (Figure 2). A repeated-measures ANOVA confirmed that there was no significant effect of the intervention on total bacterial counts (see Supplementary Table 1 for full statistical results).

### Microbiome profiling analysis–QMP

**Figure 3.**
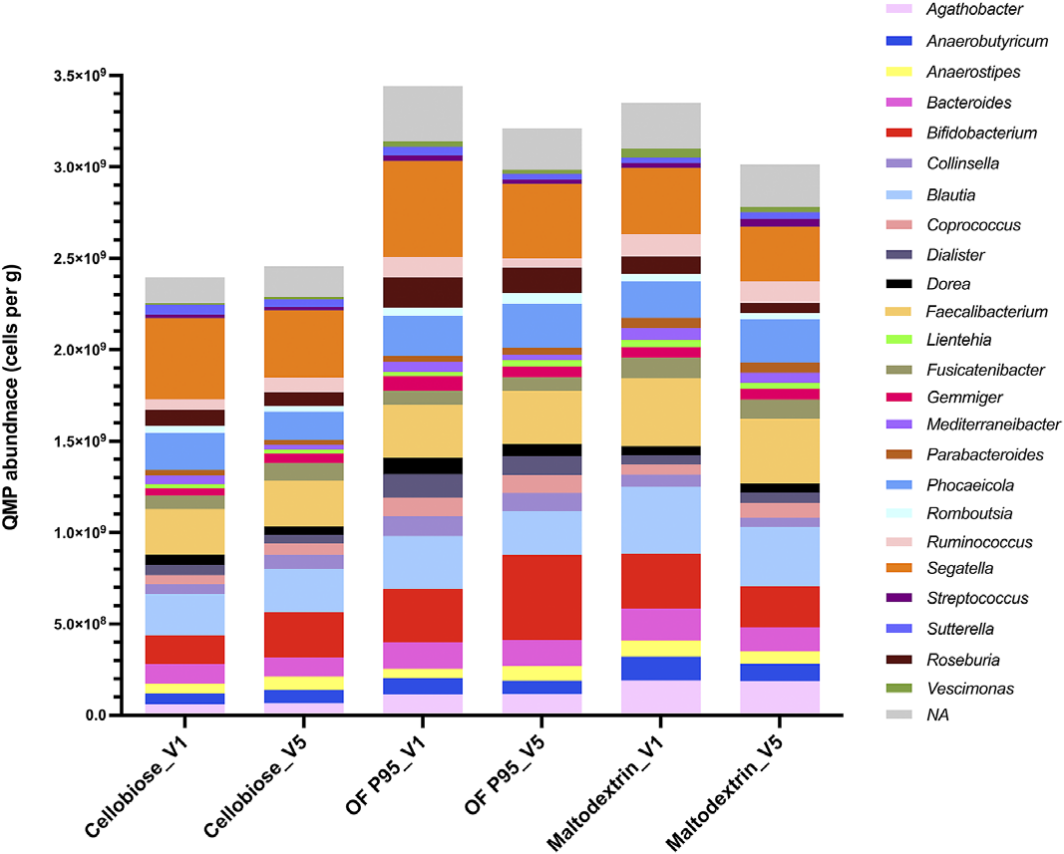
Quantitative microbiome profile (QMP) based on 16S rRNA sequencing across all interventions at V1 and V5. Numbers are expressed as cells/g faeces. Figure 3. long description.

The core microbiome was defined as genera present in at least 50% of the 37 participants at either baseline (Visit 1) or the end of the intervention (Visit 5). The mean absolute abundances of the 25 most abundant genera at baseline are presented for each intervention at both time points (Figure 3). Overall, there were no significant changes in the composition of the core microbiome from baseline to the end of the intervention in any of the three groups.

**Figure 4.**
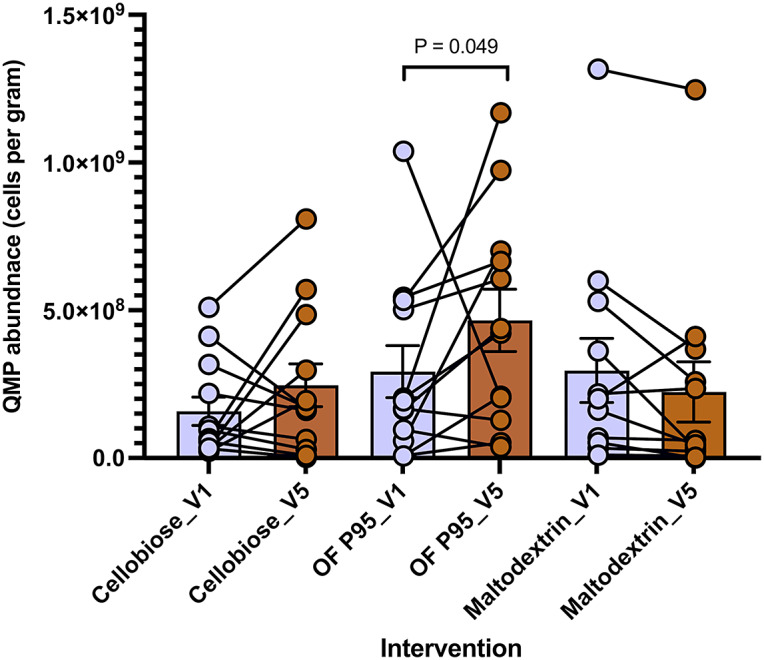
Quantitative microbiome profile differences in *Bifidobacterium* based on 16S rRNA sequencing at baseline (V1) and completion (V5) of the intervention in 37 adults. Individual, mean, and standard error (SE) were presented and expressed as numbers of cells/g faeces. Repeated-measure ANOVA was used to calculate intervention effect. Results that are statistically significant between interventions are displayed by specified *P* values. Abbreviation: OF P95, Oligofructose P95. Figure 4. long description.

The effects of the interventions on major phyla were assessed. Repeated-measures ANOVA revealed no significant changes from baseline to the end of the intervention in either the cellobiose or maltodextrin groups. However, the OF P95 group showed a significant decrease in two phyla: Euryarchaeota (*P* = 0.05) and Pseudomonadota (*P* = 0.021). A full statistical analysis is available in Supplementary Table 2. At the genus level, responses to the interventions varied. In line with the primary outcome, Figure 4 showed that the abundance of *Bifidobacterium* was significantly increased from baseline to the end of the intervention in the OF P95 group (*P* = 0.049). No significant changes in *Bifidobacterium* or *Lactobacillus* were observed in the cellobiose or maltodextrin groups. Full statistical results are available in Supplementary Table 3.

As shown in Figure 3, on an aggregate level, genus-level QMP abundance remained relatively stable across timepoints; however, underlying individual responses to the interventions were highly heterogeneous, with some participants exhibiting large increases in specific genera while others remained unchanged or declined. To account for this variability and control for baseline differences and sex, a two-way ANCOVA was performed (factors: intervention, gender; covariate: V1; dependent variable: V5). Baseline abundance was a strong predictor of follow-up levels (F(1,30) = 21.44, *P* < 0.001, partial *η*^2^ = 0.42), and sex had a modest but significant effect (F(1,30) = 5.01, *P* = 0.033, *η*^2^ = 0.14). The main effect of intervention was borderline (F(2,30) = 3.31, *P* = 0.050, *η*^2^ = 0.18), and Bonferroni-adjusted pairwise comparisons between cellobiose, OF P95, and maltodextrin were all non-significant (*P* > 0.05), although OF P95 tended to exceed maltodextrin (mean difference = 2.54 × 10^8^, *P* = 0.053).

The ANCOVA residuals for *Lactobacillus* abundance remained non-normal even after log-transformation (Shapiro–Wilk *P* = 0.006), necessitating the use of non-parametric analysis. At the end of the intervention (Visit 5), the median *Lactobacillus* abundance for all participants was 1.18 × 10^7^ cells/g (interquartile range: 6.33 × 10^6^–3.01 × 10^7^). A Kruskal–Wallis H test found no significant difference in the distribution of *Lactobacillus* abundance across the three intervention groups (H(2) = 1.19, *P* = 0.551). A median test likewise showed no difference in the proportion of participants above vs. below the overall median (χ^2^(2) = 0.999, *P* = 0.607). These results indicate that post-treatment *Lactobacillus* abundance did not differ among the cellobiose, OF P95, and maltodextrin groups.

**Figure 5.**
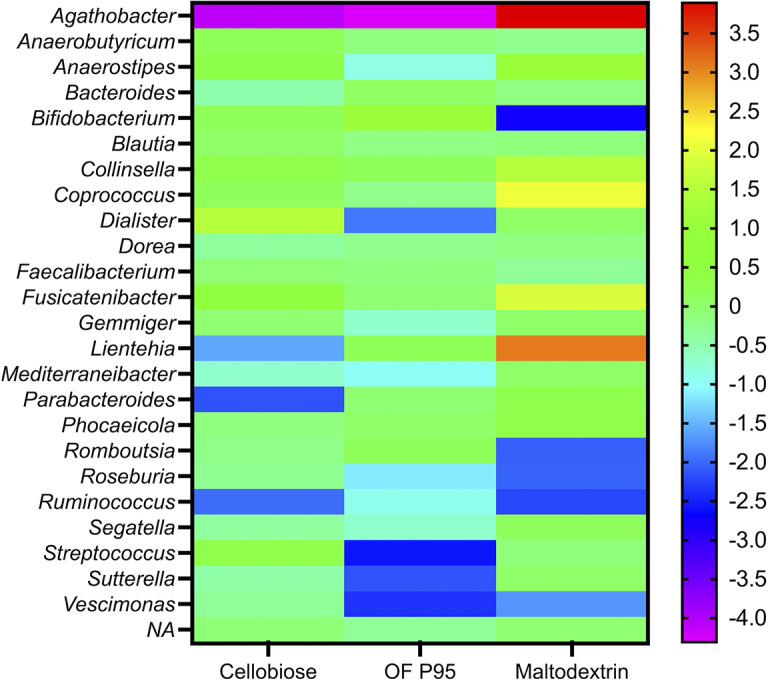
Heatmap of mean log₂ fold-change in top 25 selected genus level for each arm across the full cohort (*n* = 37). Log₂ fold-change values range from −4 (strong negative) to +4 (strong positive); colour intensity reflects the magnitude and direction (purple = negative, red = positive). Abbreviations: OF P95, oligofructose P95; NA, not assigned at the sequencing level. Figure 5. long description.

**Figure 6.**
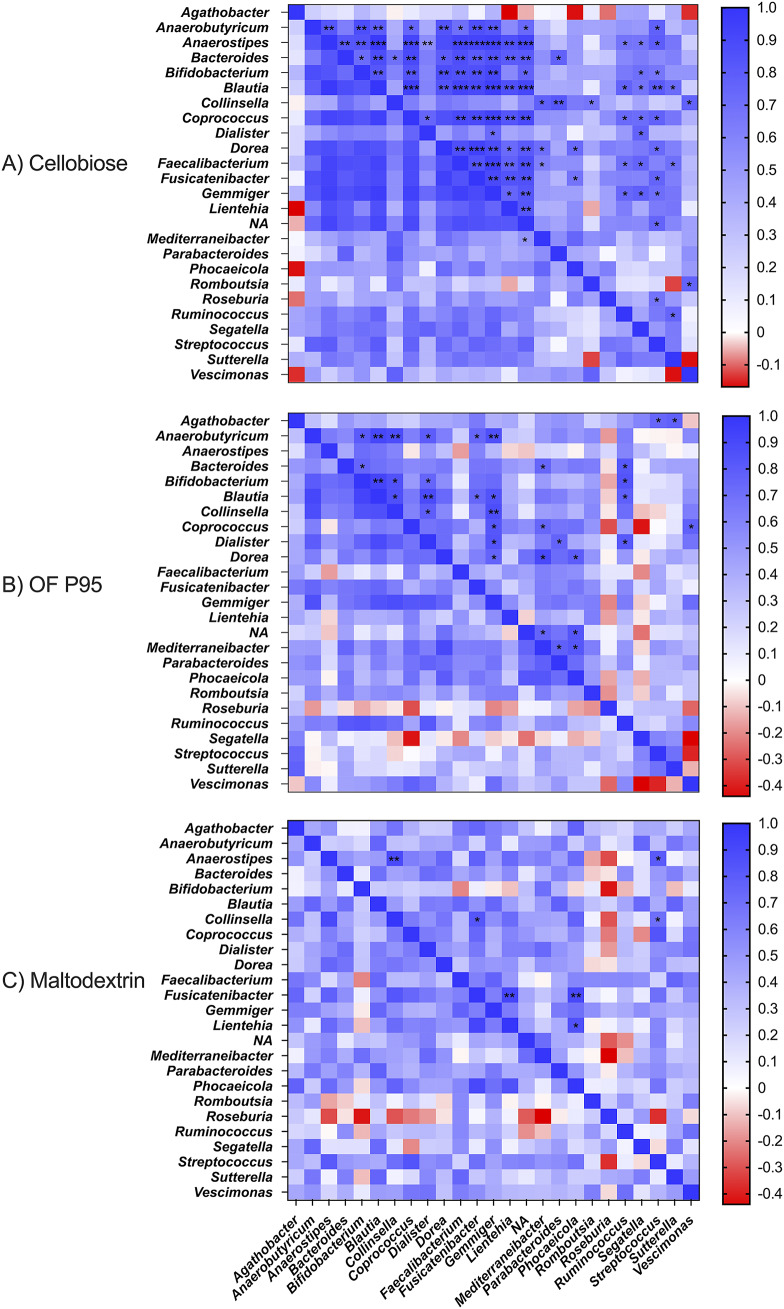
Heatmap of Spearman genus–genus correlations within each intervention. Cell colour reflects correlation coefficient (blue = positive, red = negative). Asterisks (*P ≤ 0.5; **P ≤ 0.01; ***P ≤ 0.001) denote correlations significant after BH-FDR correction; only the upper triangle of each symmetric matrix is annotated to avoid duplication. Abbreviations: OF P95, oligofructose P95; NA, not assigned at the sequencing level. Figure 6. long description.

Besides major bacterial taxa, the magnitude and pattern of overall gut-microbiota shift from V1 to V5 was also investigated. Log₂-fold-changes (V5/V1) were calculated and plotted in a heatmap (Figure 5) for each genus within each arm, they were compared across arms using ANOVA with FDR correction. Overall stability was observed across the core community: 17 of the 25 genera exhibited mean log₂-fold-changes between −2 and +2 in all three arms, corresponding to less than a twofold shift in abundance. None of the 25 genera show a statistically significant difference in mean Log₂-fold-changes across arms (adjusted p-values [q-FDR] > 0.05). To further understand the correlations between these changes, a correlation heatmap was generated among the top 25 genera, grouping by each intervention, as an exploratory, hypothesis-generating analysis. Figure 6 and Supplementary Table 4 show the genus-genus correlation matrix. In the cellobiose group, strong positive correlations (ρ > 0.6) were widespread. *Anaerostipes* formed the most links, correlating positively with 12 of the top 25 genera, nearly half of the network. Its strongest associations were with *Blautia* (Spearman’s ρ = 0.98, *P* = 9.3 $\times 10^{-6}$ ), *Coprococcus* (Spearman’s ρ = 0.92, *P* = 7.1 $\times 10^{-4}$ ), *Faecalibacterium* (Spearman’s ρ = 0.92, *P* = 5.6 $\times 10^{-4}$ ), *Fusicatenibacter* (Spearman’s ρ = 0.92, *P* = 5.6 $\times 10^{-4}$ ), and *Gemmiger* (Spearman’s ρ = 0.93, *P* = 3.0 $\times 10^{-4}$ ). *Bifidobacterium* was also found to be positively correlated with *Blautia* (Spearman’s ρ = 0.83, *P* = 0.006), *Coprococcus* (Spearman’s ρ = 0.84, *P* = 0.005), *Dorea* (Spearman’s ρ = 0.87, *P* = 0.002), *Faecalibacterium* (Spearman’s ρ = 0.85, *P* = 0.003), *Fusicatenibacter* (Spearman’s ρ = 0.86, *P* = 0.003), *Gemmiger* (Spearman’s ρ = 0.90, *P* = 0.001), *Segatella* (Spearman’s ρ = 0.70, *P* = 0.031), and *Streptococcus* (Spearman’s ρ = 0.67, *P* = 0.050). Conversely, OF P95 produced a fragmented network, *Anaerobutyricum* was found to be the most connected, correlating positively with *Bifidobacterium* (Spearman’s ρ = 0.80, *P* = 0.027), *Blautia* (Spearman’s ρ = 0.91, *P* = 0.006), *Collinsella* (Spearman’s ρ = 0.88, *P* = 0.009), *Dialister* (Spearman’s ρ = 0.79, *P* = 0.033), *Fusicatenibacter* (Spearman’s ρ = 0.74, *P* = 0.050), and *Gemmiger* (Spearman’s ρ = 0.86, *P* = 0.013). While the maltodextrin group maintained only a few significantly positive correlations, reflecting a baseline community structure.

### SCFA quantification

**Figure 7.**
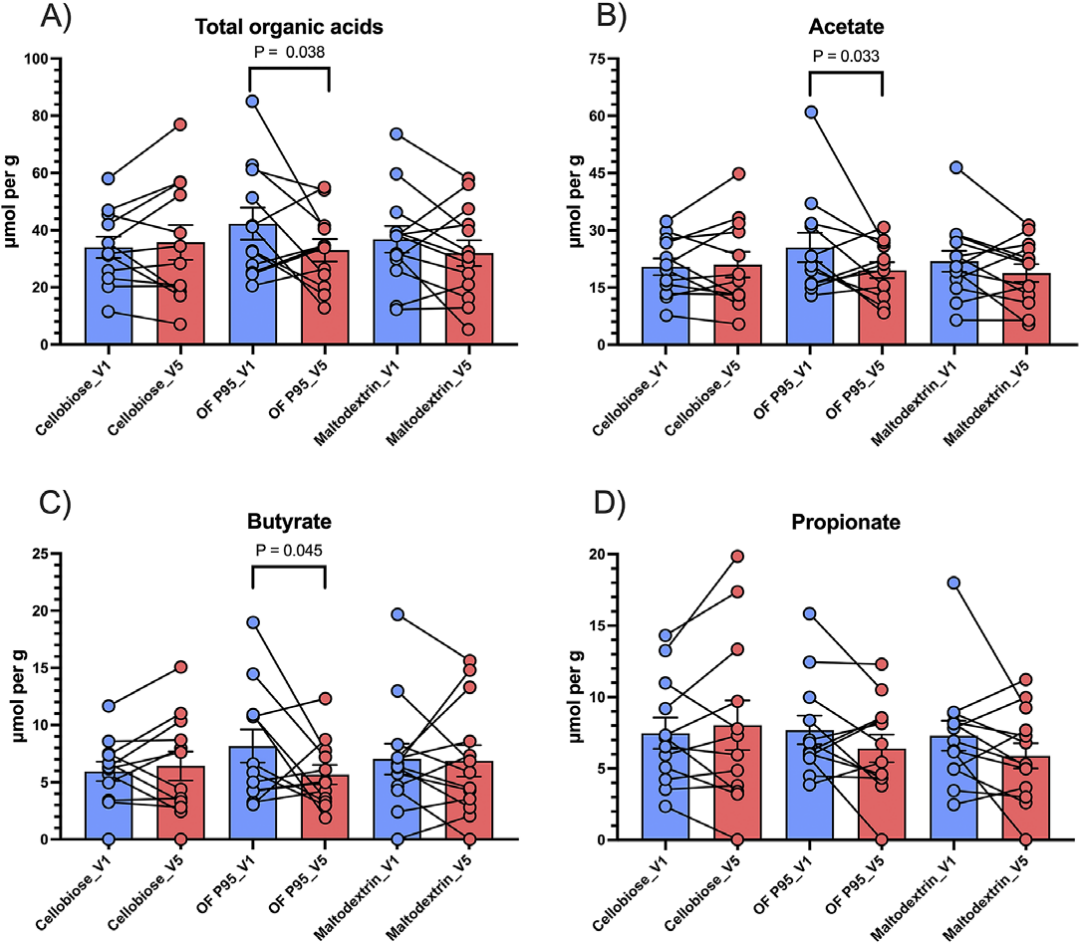
Amount of total organic acids (A), acetate (B), butyrate (C) and propionate (D) from fresh wet faeces measured by gas chromatography at V1 and V5. Individual, mean and SE were presented and expressed as numbers of μmol/g faeces. Results that are statistically significant within respective treatments are displayed by specified P-values. Error bars represent SE. Abbreviations: OF P95, Oligofructose P95. Figure 7. long description.

Metabolic profiles of faecal samples across three interventions at baseline and completion were analysed. Figure 7 and Supplementary Table 5 summarize the concentrations of total organic acids, acetate, butyrate, and propionate. Organic acids were unchanged from V1 to V5 in cellobiose and maltodextrin (*P* > 0.05), while OF P95 induced a significant decrease (*P* = 0.038). Acetate and butyrate showed no significant differences over time in cellobiose and maltodextrin (*P* > 0.05). Propionate levels remained stable with no significant changes over time across all intervention groups. Crucially, when comparing the net changes from baseline between the different groups, no statistically significant differences were observed for any of the analysed metabolites.

OF P95 again showed a significance decrease over time in acetate (*P* = 0.038) and butyrate (*P* = 0.045). Propionate showed no significant differences over time in both interventions. However, between-group comparisons of change from baseline were not significant.

In short, neither total nor individual SCFAs were significantly affected by cellobiose and maltodextrin, while OF P95 exhibited a significant decrease in total SCFAs and key organic acids. The individual subject trajectories revealed a split: most subjects show an increase in total SCFA (and in each of acetate, propionate, and butyrate) in the cellobiose group, while most subjects induced a decrease in those same SCFA in OF P95 and maltodextrin.

### Correlations between bacteriology and SCFA quantification

**Figure 8.**
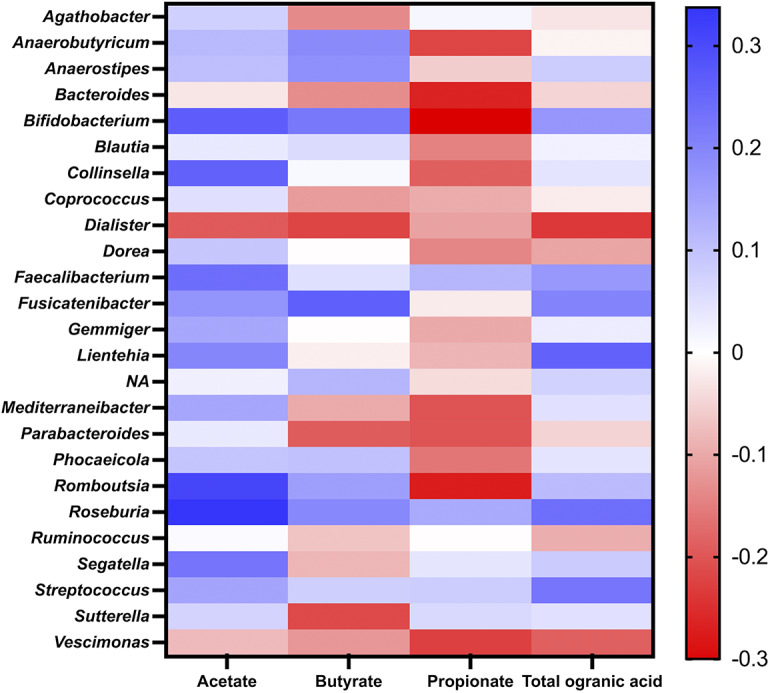
Heatmap of Spearman’s rank correlations between bacterial genera and SCFA log₂–fold-changes across the full cohort (n = 37). Spearman’s ρ (two-sided) was calculated for each genus–SCFA pair and adjusted for multiple testing with the Benjamini–Hochberg FDR. ρ values range from −0.3 (strong negative) to +0.3 (strong positive); colour intensity reflects the magnitude and direction of ρ (blue = negative, red = positive). Asterisks denote correlations surviving FDR (q < 0.05). Abbreviations: NA, not assigned at the sequencing level. Figure 8. long description.

**Figure 9.**
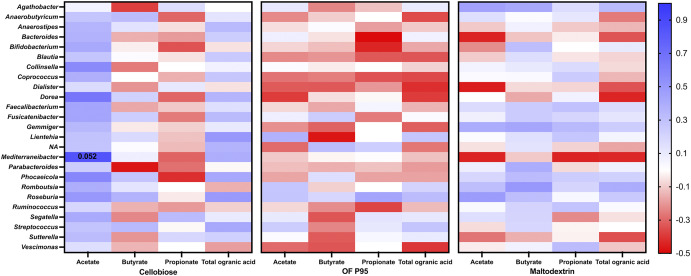
Heatmap of Spearman’s rank correlations between bacterial genera and SCFA log₂-fold-changes across the full cohort (n = 37), grouped by intervention. Spearman’s ρ (two-sided) was calculated for each genus–SCFA pair and adjusted for multiple testing with the BH-FDR. ρ values range from −0.5 (strong negative) to +1 (strong positive); colour intensity reflects the magnitude and direction of ρ (blue = positive, red = negative). Specific value denotes correlations surviving FDR (q < 0.05). Abbreviations: OF P95, oligofructose P95; NA, not assigned at the sequencing level. Figure 9. long description.

To investigate the relationships between changes in taxa and SCFA quantification, a correlation matrix using the fold change [Log2 (post/pre)] for of the QMP genera and SCFA was constructed (Figure 9). The data were analysed using a non-parametric Spearman’s rank correlation analysis, further adjusted using BH-FDR. Figure 8 and Supplementary Table 6 summarize the overall correlation heatmap between SCFA and top 25 bacteria taxa. No correlation reached statistical significance after BH-FDR correction (all *q* > 0.05). But 22 out of 25 genera had positive Spearman’s ρ with acetate fold-change (mean ρ = 0.11, range −0.20 to 0.34), yet 19 of 25 genera had negative Spearman’s ρ with propionate fold-change (mean ρ = −0.1, range −0.30 to 0.14).

No genus–SCFA pair reached significance (*q* < 0.05) in any intervention. However, there are some clear trends (Figure 9); in the cellobiose group, most genera correlated positively with acetate (median ρ = +0.12). *Mediterraneibacter* had the strongest correlation with acetate (ρ = 0.85; raw *P* = 0.0005; *q* = 0.052). Correlations with butyrate and propionate were weak and variable (median |ρ| < 0.05). In OF P95, genera tended to correlate negatively with butyrate (median ρ = −0.18) and propionate (median ρ = −0.15), mirroring the overall decline in these SCFAs despite increased bacterial counts. Acetate correlations were small (median ρ = −0.02). Last but not the least, in the maltodextrin group, correlations clustered around zero for all SCFAs (median ρ ≈ 0.04), in line with minimal changes in both bacterial abundance and SCFA levels.

## Discussion

This randomised, controlled trial used QMP and a pre-specified analysis plan to test three carbohydrate interventions, cellobiose, oligofructose P95 (OF P95), and maltodextrin. The primary endpoints were *Bifidobacterium* and *Lactobacillus* absolute abundances. Neither genus changed significantly from baseline after cellobiose consumption. In contrast, OF P95 produced a borderline increase in *Bifidobacterium*, consistent with its well-documented bifidogenic activity (Kolida et al., 2002; Meyer and Stasse-Wolthuis, 2009; Jackson et al., 2023; Van Harsselaar et al., 2024), while maltodextrin had no clear effect. Secondary analyses of overall composition showed only small, non-significant differences between interventions. Exploratory co-abundance networks revealed intervention-specific patterns. Cellobiose was associated with dense, positive correlations among butyrate-linked genera, *Anaerobutyricum*, *Anaerostipes*, and *Coprococcus*, with *Blautia* positioned as a major acetate producer (Shetty et al., 2020; Hosomi et al., 2022; Sheridan et al., 2022; Notting et al., 2023). *Bifidobacterium* clustered alongside *Gemmiger*, *Faecalibacterium*, and *Dorea* (strong positive ρ). This pattern fits known cross-feeding relationships between bifidobacteria (lactate producers) and butyrate producers (lactate consumers) (Duncan et al., 2004; Belenguer et al., 2006; Vital et al., 2014). In contrast, OF P95 networks were more fragmented, and maltodextrin largely preserved baseline structure. The correlation between *Mediterraneibacter* log₂-fold-change and acetate under cellobiose was strong but borderline. Furthermore, it is worth noting that maltodextrin, while utilized as a placebo control in this trial, may not be entirely inert. Emerging evidence indicates that maltodextrin can subtly modulate gut microbial composition and metabolic pathways (Almutairi et al., 2022), a factor that should be kept in mind when evaluating comparative baseline-to-endpoint changes between the groups.

As for the tolerability and safety, GI symptoms and stool measures were similar across interventions, indicating good tolerability of the tested cellobiose at 5–10 g/day. This result is supported by a previous human trial where cellobiose was shown to be highly tolerable, with single doses of 20 g causing no issues and repeated 15 g doses causing only minor, temporary symptoms (Moré et al., 2020). Compared with the broader prebiotic literature, inulin-type fructans/oligofructose show a dose-dependent GI profile, gas/bloating rise above ~10 g/day, although typical prebiotic doses (≈5–10 g/day) are generally acceptable, placing our findings within expected tolerability bands for fermentable carbohydrates (Kolida and Gibson, 2007; Bonnema et al., 2010; Le Bourgot et al., 2022).

Given that fructan-type substrates are preferentially fermented by bifidobacteria, the observed bifidogenic effect of OF P95 is expected. The lack of a *Lactobacillus* response is also expected, as *Lactobacillus* is typically a minor member of adult faecal communities and is more characteristic of the upper gut (Heeney et al., 2018; Jensen et al., 2023); hence, it is often hard to detect stable increases in stool collection studies. These taxa are also strain-specific and often respond to different carbohydrate niches than fructans (Kaplan and Hutkins, 2000; Walton et al., 2012; Zúñiga et al., 2018; Zheng et al., 2020; Huang et al., 2022). In short, the data suggest that while OF P95 was specifically bifidogenic, the effect of cellobiose on *Bifidobacterium* was not statistically significant. Instead, cellobiose appears to alter community interactions among various butyrate-producing bacteria rather than driving a large shift in any single genus.

A strength of this study is the use of QMP to report absolute counts (cells/g faeces) rather than relative abundances. Percentages can be misleading when increases in one taxon mathematically lower others’ percentages (Gloor et al., 2017). This method has been used for linking community variation to microbial load (Vandeputte et al., 2017). To improve the interpretability of our data, we focused our analysis on a predefined group of core genera that were prevalent across most participants. This approach reduces the statistical noise from rare taxa, which can show misleadingly large fold-changes even if they are only present in one or two individuals (Weiss et al., 2017).

This study’s ability to detect genus-level microbial shifts was likely limited by its brief duration and the use of modest doses (5–10 g/day). These factors, combined with a relatively small sample size (*n* = 37), mean that more subtle or strain-specific changes may have been missed. Furthermore, while participants remained on their normal diets to improve the external validity of the findings, this introduced dietary variability as a confounding factor that could have masked smaller prebiotic effects.

The exploratory multi-taxon analyses involved many statistical comparisons, which increases the likelihood of finding false positives by chance, even after applying a FDR correction. To address this, our interpretation strategy prioritized biologically meaningful signals over simple statistical significance. We therefore focused on the magnitude of change (effect sizes) and consistent trends across related bacterial groups, treating these exploratory results as hypothesis-generating findings for future research.

Finally, QMP provides stool-based absolute counts and therefore characterises luminal communities; it does not capture the mucosa-associated microbiota and cannot localise changes to specific colonic regions; conclusions are therefore restricted to stool. Likewise, faecal SCFA concentrations are conservative endpoints: because most SCFA are absorbed and metabolised in the large intestine, stool levels reflect the residual rather than the production rate and are sensitive to transit and dilution effects.

In summary, this *in vivo* intervention trial confirmed the selective fermentation of OF P95 by *Bifidobacterium* and suggested that cellobiose may reshape co-abundance patterns among butyrate-linked genera. The core community remained stable in absolute terms. While no statistically significant differences were observed between the intervention groups, the data suggest a trend towards an increase in beneficial bacteria following cellobiose consumption.

We did not find major shifts in total microbial numbers or endpoint metabolite levels. However, this does not mean cellobiose has no biological activity. These results show the limits of testing a healthy, resilient gut using only static “before and after” measurements, which can easily miss fluid ecosystem changes happening during the intervention. Because a single human study does not give a definitive result, continuous *in vivo* testing is necessary to consistently validate these ingredients. Future human trials must inevitably continue to rely on robust pre- and post-intervention designs to provide essential clinical data.

To complement these essential human trials and better validate underlying mechanisms, future work can pair *in vivo* studies with dynamic *in vitro* gut models, such as continuous fermentation systems. These laboratory systems offer a controlled platform to track rapid community interactions and metabolic fluxes in real time. Using *in vitro* tools to resolve these transient pathways will provide key mechanistic insights that can then be used to better design and validate subsequent, fully powered human interventions.

Nevertheless, these proof-of-concept data from our human trial indicate that cellobiose is well-tolerated and shows a promising trend towards modulating the gut microbiota.

Establishing the definitive prebiotic status of cellobiose will require a broader body of evidence, as outlined by consensus panels (Gibson et al., 2017; Hutkins et al., 2024). Future work should also build on the findings of this study by employing larger, longer-duration clinical trials with predefined health endpoints. Furthermore, applying methods such as shotgun metagenomics and metabolomics in future trials would allow for a mechanism analysis, directly linking specific microbial genes to the metabolic products of cellobiose fermentation in the human gut.

## Supporting information

10.1017/gmb.2026.10032.sm001 Huang et al. supplementary material Huang et al. supplementary material

## Data Availability

Processed data supporting the findings of this article will be publicly deposited in the University of Reading Research Data Archive upon acceptance for publication. The data are fully de-identified to comply with ethical and privacy guidelines and the Helsinki Declaration. The data are currently available from the corresponding author, R.A.R., upon reasonable request and for peer review purposes. The study protocol was pre-registered at ClinicalTrials.gov (Identifier: NCT07097389) and the full protocol document is provided within the Supplementary Material accompanying this article.

## Supplementary material

The supplementary material for this article can be found at http://doi.org/10.1017/gmb.2026.10032.

## Long descriptions

### Table 1. Long description

The table consists of four columns titled Probes, Sequence (5 prime to 3 prime), Targeted groups, and Reference.

* Row 1: Non Eub. Sequence: A C T C C T A C G G G A G G C A G C. Targeted groups: Control probe complementary to E U B 3 3 8; non bacteria. Reference: Wallner et al. 1993.

* Row 2: Eub 338 I. Sequence: G C T G C C T C C C G T A G G A G T. Targeted groups: Most bacteria. Reference: Amann et al. 1990.

* Row 3: Eub 338 I-I I. Sequence: G C A G C C A C C C G T A G G T G T. Targeted groups: Most bacteria. Reference: Daims et al. 1999.

* Row 4: Eub 338 I-I I-I I I. Sequence: G C T G C C A C C C G T A G G T G T. Targeted groups: Most bacteria. Reference: Daims et al. 1999.

Navigate back to Table 1..

### Figure 1. Long description

The flowchart is organized into four vertical stages.

1. Enrolment phase: At the top, 44 participants were assessed for eligibility. 39 were excluded: 3 for not meeting inclusion criteria, 1 declined, and 1 for other reasons. This left 39 participants who were randomized.

2. Allocation phase: The 39 participants were split into three groups.

* Left: 13 participants allocated to intervention D-Cellobiose; all 13 received the intervention.

* Center: 12 participants allocated to intervention W-O F P 95; all 12 received the intervention.

* Right: 14 participants allocated to intervention R-Maltodextrin; all 14 received the intervention.

3. Follow-Up phase:

* D-Cellobiose group: 1 discontinued due to sample collection issues and 1 was lost to follow-up for the primary outcome due to sample collection issues.

* W-OF P 95 group: 0 discontinued and 0 were lost to follow-up.

* R-Maltodextrin group: 1 discontinued due to frequent bowel movements and 1 was lost to follow-up due to an adverse event.

4. Analysis phase:

* D-Cellobiose group: 12 analyzed for primary outcome; 0 excluded.

* W-OF P 95 group: 12 analyzed for primary outcome; 0 excluded.

* R-Maltodextrin group: 13 analyzed for primary outcome; 0 excluded.

Navigate back to Figure 1..

### Table 2. Long description

The table consists of five columns: Characteristic, Cellobiose (n = 12), O F P 95 (n = 12), Maltodextrin (n = 13), and Total (n = 37).

Data rows are as follows:

* Age (y): Cellobiose is 33.8 plus or minus 17.2 (range 22 to 72). O F P 95 is 37.1 plus or minus 15.2 (range 20 to 82). Maltodextrin is 34.1 plus or minus 12.0 (range 20 to 59). Total is 35.0 plus or minus 14.6 (range 20 to 82).

* Male, n (%): Cellobiose is 3 (25%). O F P 95 is 6 (50%). Maltodextrin is 5 (38.5%). Total is 14 (37.8%).

* Female, n (%): Cellobiose is 9 (75%). O F P 95 is 6 (50%). Maltodextrin is 8 (61.5%). Total is 23 (62.2%).

Note: Age data are presented as standard error S E (range). O F P 95 stands for oligofructose P 95.

Navigate back to Table 2..

### Figure 2. Long description

The Y axis is labeled log 10 cells forward slash g wet faeces, ranging from 8.0 to 10.0. The X axis is labeled Intervention and contains six categories.

From left to right, the bars represent:

* Cellobiose underscore V 1: Light blue bar with light blue circles. Mean is approximately 9.4 with an S E error bar extending from 9.3 to 9.5. Individual points range from 8.7 to 9.8.

* Cellobiose underscore V 5: Brown bar with orange triangles. Mean is approximately 9.35 with an S E error bar from 9.25 to 9.5. Individual points range from 8.3 to 9.8.

* O F P 95 underscore V 1: Light blue bar with light blue circles. Mean is approximately 9.6 with an S E error bar from 9.5 to 9.7. Individual points range from 9.1 to 9.9.

* O F P 95 underscore V 5: Brown bar with orange triangles. Mean is approximately 9.6 with an S E error bar from 9.5 to 9.7. Individual points range from 9.2 to 9.9.

* Maltodextrin underscore V 1: Light blue bar with light blue circles. Mean is approximately 9.5 with an S E error bar from 9.4 to 9.6. Individual points range from 9.1 to 9.9.

* Maltodextrin underscore V 5: Brown bar with orange triangles. Mean is approximately 9.5 with an S E error bar from 9.4 to 9.6. Individual points range from 9.0 to 9.9.

All bars show relatively high bacterial density with overlapping error bars, indicating similar mean values across the different interventions and time points.

Navigate back to Figure 2..

### Figure 3. Long description

A stacked bar chart with a Y-axis labeled Q M P abundance (cells per g) ranging from 0.0 to 3.5 times 10 super 9. The X-axis lists six intervention groups: Cellobiose_V 1, Cellobiose_V 5, O F P 95_V 1, O F P 95_V 5, Maltodextrin_V 1, and Maltodextrin_V 5.

Each bar is composed of colored segments representing different bacterial genera. From bottom to top, the primary segments include:

* Agathobacter (light purple)

* Anaerobutyricum (dark blue)

* Anaerostipes (yellow)

* Bacteroides (pink)

* Bifidobacterium (red)

* Blautia (light blue)

* Faecalibacterium (tan)

* Phocaeicola (medium blue)

* Segatella (orange)

* N A (grey) at the top of every bar.

Key trends:

* Cellobiose groups show the lowest total abundance, approximately 2.4 to 2.5 times 10 super 9.

* O F P 95_V 1 shows the highest total abundance, reaching nearly 3.5 times 10 super 9.

* Bifidobacterium (red) and Blautia (light blue) segments are notably larger in the O F P 95 and Maltodextrin groups compared to Cellobiose.

* Segatella (orange) remains a dominant segment across all interventions, consistently positioned near the top of the colored stack.

Navigate back to Figure 3..

### Figure 4. Long description

A vertical bar graph with overlaid individual data points and connecting lines.

Axes:

* The Y-axis is labeled QMP abundance (cells per gram) with a scale from 0.0 to 1.5 times 10 super 9.

* The X-axis is labeled Intervention and contains three paired groups: Cellobiose, O F P95, and Maltodextrin.

Data Groups from left to right:

* Cellobiose: The light blue bar for V1 is approximately 0.2 times 10 super 9. The brown bar for V5 shows a slight increase to approximately 0.25 times 10 super 9. Individual lines show varied trends with some sharp increases.

* OF P95: The light blue bar for V1 is approximately 0.3 times 10 super 9. The brown bar for V5 increases to nearly 0.5 times 10 super 9. A bracket above these two bars indicates a statistically significant difference with P equals 0.049. Several individual lines show steep upward trajectories.

* Maltodextrin: The light blue bar for V1 is approximately 0.3 times 10 super 9. The brown bar for V5 shows a slight decrease to approximately 0.25 times 10 super 9. Most individual lines trend downward or remain flat.

Visual Elements:

* Light blue bars and circles represent baseline V1.

* Brown bars and circles represent completion V5.

* Error bars indicate standard error S E.

* Lines connect individual data points between V1 and V5 for each participant.

Navigate back to Figure 4..

### Figure 5. Long description

A heatmap grid with 25 rows of bacterial genera and 3 columns representing treatment arms.

* Axes and Scale: The Y-axis lists genera including Agathobacter, Anaerobutyricum, Bifidobacterium, and others, ending with N A. The X-axis lists Cellobiose, O F P 95, and Maltodextrin. A vertical color bar on the right indicates log sub 2 fold-change from minus 4 (purple) to 0 (light green) to plus 4 (red).

* Data Trends:

* Agathobacter shows a strong negative fold-change (purple) in Cellobiose and OF P 95 but a strong positive fold-change (red) in Maltodextrin.

* Bifidobacterium shows a distinct negative fold-change (dark blue) specifically in the Maltodextrin arm.

* Lientehia shows a strong positive fold-change (orange-red) in the Maltodextrin arm.

* Streptococcus, Sutterella, and Vescimonas show strong negative fold-changes (blue) primarily in the OF P 95 arm.

* Parabacteroides and Ruminococcus show negative fold-changes in the Cellobiose arm.

* Most other genera, such as Anaerostipes and Blautia, maintain values near 0 (light green) across all three arms.

Navigate back to Figure 5..

### Figure 6. Long description

A multi-panel figure with three heatmaps labeled A, B, and C. Each heatmap shares a common Y-axis on the left and X-axis at the bottom listing 26 bacterial genera including Agathobacter, Anaerobutyricum, Anaerostipes, Bacteroides, Bifidobacterium, Blautia, Collinsella, Coprococcus, Dialister, Dorea, Faecalibacterium, Fusicatenibacter, Gemmiger, Lientehia, N A, Mediterraneibacter, Parabacteroides, Phocaeicola, Romboutsia, Roseburia, Ruminococcus, Segatella, Streptococcus, Sutterella, and Vescimonas. A color scale bar to the right of each plot ranges from negative 0.4 (dark red) through 0 (white) to 1.0 (dark blue).

* Panel A, Cellobiose: Shows a dense cluster of dark blue cells in the upper-left quadrant, particularly among Anaerobutyricum, Anaerostipes, Bacteroides, and Bifidobacterium. This area is heavily annotated with asterisks indicating high statistical significance (P less than or equal to 0.001). The lower-right quadrant shows more varied correlations with scattered red cells for Sutterella and Vescimonas.

* Panel B, O F P95: Displays a more diffuse correlation pattern compared to panel A. Significant positive correlations (blue with asterisks) are visible between Agathobacter and Vescimonas, and between Bifidobacterium and Blautia. Red cells indicating negative correlations are more prominent, specifically involving Segatella and Roseburia.

* Panel C, Maltodextrin: Shows the least amount of significant correlations. The heatmap is dominated by light blue and white cells. Notable negative correlations (red) are seen for Roseburia against several other genera and for N A against Mediterraneibacter. Significant positive correlations are sparse, with a few marked in the upper-left and center-right sections.

Navigate back to Figure 6..

### Figure 7. Long description

A four-panel set of bar graphs with overlaid individual data points and connecting lines. Each graph shares a common x-axis with three treatment pairs: Cellobiose underscore V 1 and V 5, O F P 95 underscore V 1 and V 5, and Maltodextrin underscore V1 and V5. Blue bars represent V 1 and red bars represent V 5. The y-axis for all panels is mu mol per g.

Panel A, Total organic acids: The y-axis ranges from 0 to 100. Cellobiose shows a slight increase from approximately 34 to 36. OF P 95 shows a significant decrease from 42 to 33 with a P-value equal to 0.038. Maltodextrin shows a decrease from 37 to 32.

Panel B, Acetate: The y-axis ranges from 0 to 75. Cellobiose remains stable around 20. OF P 95 shows a significant decrease from 25 to 19 with a P-value equal to 0.033. Maltodextrin decreases from 22 to 18.

Panel C, Butyrate: The y-axis ranges from 0 to 25. Cellobiose shows a slight increase from 6 to 6.5. O F P 95 shows a significant decrease from 8 to 5.5 with a P-value equal to 0.045. Maltodextrin remains stable around 7.

Panel D, Propionate: The y-axis ranges from 0 to 20. Cellobiose increases from 7.5 to 8. OF P 95 decreases from 7.5 to 6. Maltodextrin decreases from 7 to 6. No significant P-values are noted for Panel D.

In all panels, individual data points are connected by lines to show the trajectory of change for each subject between V 1 and V 5. Error bars represent S E.

Navigate back to Figure 7..

### Figure 8. Long description

A rectangular heatmap grid with 24 rows of bacterial genera and 4 columns of short-chain fatty acids.

Axes and Scale:

- Y-axis (left): Lists genera including Agathobacter, Anaerobutyricum, Anaerostipes, Bacteroides, Bifidobacterium, Blautia, Collinsella, Coprococcus, Dialister, Dorea, Faecalibacterium, Fusicatenibacter, Gemmiger, Lientehia, N A, Mediterraneibacter, Parabacteroides, Phocaeicola, Romboutsia, Roseburia, Ruminococcus, Segatella, Streptococcus, Sutterella, and Vescimonas.

- X-axis (bottom): Acetate, Butyrate, Propionate, and Total organic acid.

- Color Scale (right): A vertical bar ranging from +0.3 (dark blue) at the top, through 0 (white) in the middle, to -0.3 (dark red) at the bottom.

Data Distribution:

- Acetate column: Shows predominantly blue (positive) correlations, with the strongest blue intensities at Bifidobacterium, Collinsella, Romboutsia, and Roseburia. Dialister and Vescimonas show red (negative) correlations.

- Butyrate column: Displays a mix of light blue and light red. Dialister and Sutterella show the most distinct red (negative) correlations.

- Propionate column: Features the most prominent red (negative) clusters, particularly for Anaerobutyricum, Bacteroides, Bifidobacterium, Mediterraneibacter, Parabacteroides, and Romboutsia.

- Total organic acid column: Shows generally weaker correlations (lighter shades), with Dialister and Vescimonas leaning red, while Lientehia and Streptococcus lean blue.

Navigate back to Figure 8..

### Figure 9. Long description

The heatmap is divided into three panels from left to right: Cellobiose, O F P95, and Maltodextrin. The Y-axis lists 25 bacterial genera: Agathobacter, Anaerobutyricum, Anaerostipes, Bacteroides, Bifidobacterium, Blautia, Collinsella, Coprococcus, Dialister, Dorea, Faecalibacterium, Fusicatenibacter, Gemmiger, Lientehia, NA, Mediterraneibacter, Parabacteroides, Phocaeicola, Romboutsia, Roseburia, Ruminococcus, Segatella, Streptococcus, Sutterella, and Vescimonas. The X-axis for each panel lists four SCFA categories: Acetate, Butyrate, Propionate, and Total organic acid.

A color scale on the far right indicates Spearman rho values from negative 0.5 (dark red) to positive 1.0 (dark blue), with white representing zero.

* In the Cellobiose panel, the Acetate column shows a consistent positive correlation (blue) across almost all genera, with a specific value of 0.052 noted for Mediterraneibacter. The Butyrate and Propionate columns show mixed, weaker correlations with notable negative (red) clusters for Agathobacter and Parabacteroides.

* In the O F P95 panel, correlations are more fragmented. Strong negative correlations (red) are visible for Lientehia with Butyrate and Dorea with Acetate. Strong positive correlations (blue) appear for Bifidobacterium with Propionate.

* In the Maltodextrin panel, the correlations are generally weaker (lighter colors). Notable negative correlations (red) are seen for Dialister and Mediterraneibacter with Acetate and Propionate.

Navigate back to Figure 9..

## References

[r1] Almutairi R, Basson AR, Wearsh P, Cominelli F and Rodriguez-Palacios A (2022) Validity of food additive maltodextrin as placebo and effects on human gut physiology: systematic review of placebo-controlled clinical trials. European Journal of Nutrition 61(6), 2853–2871. 10.1007/s00394-022-02802-5. 35230477 PMC9835112

[r2] Amann RI, Binder BJ, Olson RJ, Chisholm SW, Devereux R and Stahl DA (1990) Combination of 16S rRNA-targeted oligonucleotide probes with flow cytometry for analyzing mixed microbial populations. Applied and Environmental Microbiology 56(6), 1919–1925. 10.1128/aem.56.6.1919-1925.1990. 2200342 PMC184531

[r3] Belenguer A, Duncan SH, Calder AG, Holtrop G, Louis P, Lobley GE and Flint HJ (2006) Two routes of metabolic cross-feeding between *Bifidobacterium adolescentis* and butyrate-producing anaerobes from the human gut. Applied and Environmental Microbiology 72(5), 3593–3599. 10.1128/AEM.72.5.3593-3599.2006. 16672507 PMC1472403

[r4] Bonnema AL, Kolberg LW, Thomas W and Slavin JL (2010) Gastrointestinal tolerance of chicory inulin products. Journal of the American Dietetic Association 110(6), 865–868. 10.1016/j.jada.2010.03.025. 20497775

[r5] Daims H, Brühl A, Amann R, Schleifer KH and Wagner M (1999) The domain-specific probe EUB338 is insufficient for the detection of all bacteria: Development and evaluation of a more comprehensive probe set. Systematic and Applied Microbiology 22(3), 434–444. 10.1016/s0723-2020(99)80053-8. 10553296

[r6] Dempsey E and Corr SC (2022) *Lactobacillus* spp. for gastrointestinal health: Current and future perspectives. Frontiers in Immunology 13, 840245. 10.3389/fimmu.2022.840245. 35464397 PMC9019120

[r7] Duncan SH, Louis P and Flint HJ (2004) Lactate-utilizing bacteria, isolated from human feces, that produce butyrate as a major fermentation product. Applied and Environmental Microbiology 70(10), 5810–5817. 10.1128/aem.70.10.5810-5817.2004. 15466518 PMC522113

[r8] Gibson GR, Hutkins R, Sanders ME, Prescott SL, Reimer RA, Salminen SJ, Scott K, Stanton C, Swanson KS, Cani PD, Verbeke K and Reid G (2017) Expert consensus document: The International Scientific Association for Probiotics and Prebiotics (ISAPP) consensus statement on the definition and scope of prebiotics. Nature Reviews. Gastroenterology & Hepatology 14(8), 491–502. 10.1038/nrgastro.2017.75. 28611480

[r9] Gloor GB, Macklaim JM, Pawlowsky-Glahn V and Egozcue JJ (2017) Microbiome datasets are compositional: and this is not optional. Frontiers in Microbiology 8, 2224. 10.3389/fmicb.2017.02224. 29187837 PMC5695134

[r10] Heeney DD, Gareau MG and Marco ML (2018) Intestinal *Lactobacillus* in health and disease, a driver or just along for the ride? Current Opinion in Biotechnology 49, 140–147. 10.1016/j.copbio.2017.08.004. 28866243 PMC5808898

[r11] Hopewell S, Chan A-W, Collins GS, Hróbjartsson A, Moher D, Schulz KF, Tunn R, Aggarwal R, Berkwits M, Berlin JA, Bhandari N, Butcher NJ, Campbell MK, Chidebe RCW, Elbourne D, Farmer A, Fergusson DA, Golub RM, Goodman SN, Hoffmann TC, Ioannidis JPA, Kahan BC, Knowles RL, Lamb SE, Lewis S, Loder E, Offringa M, Ravaud P, Richards DP, Rockhold FW, Schriger DL, Siegfried NL, Staniszewska S, Taylor RS, Thabane L, Torgerson D, Vohra S, White IR, Boutron I (2025) CONSORT 2025 statement: updated guideline for reporting randomised trials. The Lancet 405(10489), 1633–1640. 10.1016/S0140-6736(25)00672-5. 40245901

[r12] Hosomi K, Saito M, Park J, Murakami H, Shibata N, Ando M, Nagatake T, Konishi K, Ohno H, Tanisawa K, Mohsen A, Chen Y-A, Kawashima H, Natsume-Kitatani Y, Oka Y, Shimizu H, Furuta M, Tojima Y, Sawane K, Saika A, Kondo S, Yonejima Y, Takeyama H, Matsutani A, Mizuguchi K, Miyachi M and Kunisawa J (2022) Oral administration of *Blautia wexlerae* ameliorates obesity and type 2 diabetes via metabolic remodeling of the gut microbiota. Nature Communications 13(1), 4477. 10.1038/s41467-022-32015-7. PMC9388534 35982037

[r13] Hou K, Wu Z-X, Chen X-Y, Wang J-Q, Zhang D, Xiao C, Zhu D, Koya JB, Wei L, Li J and Chen Z-S (2022) Microbiota in health and diseases. Signal Transduction and Targeted Therapy 7(1), 135. 10.1038/s41392-022-00974-4. 35461318 PMC9034083

[r14] Huang R, Wu F, Zhou Q, Wei W, Yue J, Xiao B and Luo Z (2022) *Lactobacillus* and intestinal diseases: Mechanisms of action and clinical applications. Microbiological Research 260, 127019. 10.1016/j.micres.2022.127019. 35421680

[r15] Hutkins R, Walter J, Gibson GR, Bedu-Ferrari C, Scott K, Tancredi DJ, Wijeyesekera A and Sanders ME (2024) Classifying compounds as prebiotics – scientific perspectives and recommendations. Nature Reviews Gastroenterology & Hepatology. 10.1038/s41575-024-00981-6. 39358591

[r16] Jackson PPJ, Wijeyesekera A, Williams CM, Theis S, van Harsselaar J and Rastall RA (2023) Inulin-type fructans and 2’fucosyllactose alter both microbial composition and appear to alleviate stress-induced mood state in a working population compared to placebo (maltodextrin): the EFFICAD Trial, a randomized, controlled trial. The American Journal of Clinical Nutrition 118(5), 938–955. 10.1016/j.ajcnut.2023.08.016. 37657523 PMC10636234

[r17] Jensen BAH, Heyndrickx M, Jonkers D, Mackie A, Millet S, Naghibi M, Pærregaard SI, Pot B, Saulnier D, Sina C, Sterkman LGW, Van den Abbeele P, Venlet NV, Zoetendal EG and Ouwehand AC (2023) Small intestine vs. colon ecology and physiology: Why it matters in probiotic administration. Cell Reports Medicine 4(9), 101190. 10.1016/j.xcrm.2023.101190. 37683651 PMC10518632

[r18] Kaplan H and Hutkins RW (2000) Fermentation of fructooligosaccharides by lactic acid bacteria and bifidobacteria. Applied and Environmental Microbiology 66(6), 2682–2684. 10.1128/AEM.66.6.2682-2684.2000. 10831458 PMC110601

[r19] Kolida S and Gibson GR (2007) Prebiotic capacity of inulin-type fructans. The Journal of Nutrition 137(11), 2503S–2506S. 10.1093/jn/137.11.2503S. 17951493

[r20] Kolida S, Tuohy K and Gibson GR (2002) Prebiotic effects of inulin and oligofructose. The British Journal of Nutrition 87(6), 193–197. 10.1079/bjnbjn/2002537. 12088518

[r21] Le Bourgot C, Rigaudier F, Juhel C, Herpin F and Meunier C (2022) Gastrointestinal tolerance of short-chain fructo-oligosaccharides from sugar beet: an observational, connected, dose-ranging study in healthy volunteers. Nutrients 14(7), 1461. 10.3390/nu14071461. 35406074 PMC9002795

[r22] Meyer D and Stasse-Wolthuis M (2009) The bifidogenic effect of inulin and oligofructose and its consequences for gut health. European Journal of Clinical Nutrition 63(11), 1277–1289. 10.1038/ejcn.2009.64. 19690573

[r23] Moré MI, Postrach E, Bothe G, Heinritz S and Uebelhack R (2020) A dose-escalation study demonstrates the safety and tolerability of cellobiose in healthy subjects. Nutrients 12(1). 10.3390/nu12010064. PMC7019479 31881808

[r24] Negishi H, Ichikawa A, Takahashi S, Kano H and Makino S (2025) Targeted prebiotic application of gluconic acid-containing oligosaccharides promotes Faecalibacterium growth through microbial cross-feeding networks. The ISME Journal 19(1). 10.1093/ismejo/wraf027. PMC11922316 39936592

[r25] Notting F, Pirovano W, Sybesma W and Kort R (2023) The butyrate-producing and spore-forming bacterial genus *Coprococcus* as a potential biomarker for neurological disorders. Gut Microbiome 4, e16. 10.1017/gmb.2023.14. 39295905 PMC11406416

[r26] O’Callaghan A and van Sinderen D (2016) Bifidobacteria and their role as members of the human gut microbiota. Frontiers in Microbiology 7, 925. 10.3389/fmicb.2016.00925. 27379055 PMC4908950

[r27] Richardson AJ, Calder AG, Stewart CS and Smith A (1989) Simultaneous determination of volatile and non-volatile acidic fermentation products of anaerobes by capillary gas chromatography. Letters in Applied Microbiology 9(1), 5–8. 10.1111/j.1472-765X.1989.tb00278.x.

[r28] Schmidt TSB, Raes J and Bork P (2018) The human gut microbiome: from association to modulation. Cell 172(6), 1198–1215. 10.1016/j.cell.2018.02.044. 29522742

[r29] Sheridan PO, Louis P, Tsompanidou E, Shaw S, Harmsen HJ, Duncan SH, Flint HJ and Walker AW (2022) Distribution, organization and expression of genes concerned with anaerobic lactate utilization in human intestinal bacteria. Microbial Genomics 8(1). 10.1099/mgen.0.000739. PMC8914356 35077342

[r30] Shetty SA, Boeren S, Bui TPN, Smidt H and de Vos WM (2020) Unravelling lactate-acetate and sugar conversion into butyrate by intestinal Anaerobutyricum and Anaerostipes species by comparative proteogenomics. Environmental Microbiology 22(11), 4863–4875. 10.1111/1462-2920.15269. 33001550 PMC7702098

[r31] Turck D, Bohn T, Castenmiller J, De Henauw S, Hirsch-Ernst KI, Maciuk A, Mangelsdorf I, McArdle HJ, Naska A, Pelaez C, Pentieva K, Siani A, Thies F, Tsabouri S, Vinceti M, Cubadda F, Frenzel T, Heinonen M, Marchelli R, Neuhäuser-Berthold M, Poulsen M, Sanz Y, Schlatter JR, van Loveren H, Ackerl R, Gelbmann W, Steinkellner H and Knutsen HK (2022) Safety of cellobiose as a novel food pursuant to regulation (EU) 2015/2283. EFSA Journal 20(11), e07596. 10.2903/j.efsa.2022.7596. 36381122 PMC9644228

[r32] Ugwu OP, Alum EU, Okon MB and Obeagu EI (2024) Mechanisms of microbiota modulation: Implications for health, disease, and therapeutic interventions. Medicine (Baltimore) 103(19), e38088. 10.1097/md.0000000000038088. 38728472 PMC11081615

[r33] Valdes AM, Walter J, Segal E and Spector TD (2018) Role of the gut microbiota in nutrition and health. BMJ 361, k2179. 10.1136/bmj.k2179. 29899036 PMC6000740

[r34] Van Harsselaar J, Mödinger Y, Dharsono T, Menzel D, Theis S and Schön C (2024) Prebiotic effect of oligofructose after 2 weeks supplementation with a low dose: A randomized, double-blind, placebo-controlled, cross-over study. Journal of Functional Foods 119, 106356. 10.1016/j.jff.2024.106356.

[r35] Vandeputte D, Kathagen G, D’Hoe K, Vieira-Silva S, Valles-Colomer M, Sabino J, Wang J, Tito RY, De Commer L, Darzi Y, Vermeire S, Falony G and Raes J (2017) Quantitative microbiome profiling links gut community variation to microbial load. Nature 551(7681), 507–511. 10.1038/nature24460. 29143816

[r36] Vital M, Howe AC and Tiedje JM (2014) Revealing the bacterial butyrate synthesis pathways by analyzing (Meta)genomic data. MBio 5(2), e00889. 10.1128/mbio.00889-14. 24757212 PMC3994512

[r37] Wallner G, Amann R and Beisker W (1993) Optimizing fluorescent in situ hybridization with rRNA-targeted oligonucleotide probes for flow cytometric identification of microorganisms. Cytometry 14(2), 136–143. 10.1002/cyto.990140205. 7679962

[r38] Walton GE, van den Heuvel EGHM, Kosters MHW, Rastall RA, Tuohy KM and Gibson GR (2012) A randomised crossover study investigating the effects of galacto-oligosaccharides on the faecal microbiota in men and women over 50 years of age. British Journal of Nutrition 107(10), 1466–1475. 10.1017/S0007114511004697. 21910949

[r39] Weiss S, Xu ZZ, Peddada S, Amir A, Bittinger K, Gonzalez A, Lozupone C, Zaneveld JR, Vázquez-Baeza Y, Birmingham A, Hyde ER and Knight R (2017) Normalization and microbial differential abundance strategies depend upon data characteristics. Microbiome 5(1), 27. 10.1186/s40168-017-0237-y. 28253908 PMC5335496

[r40] Zafar H and Saier MH (2021) Gut Bacteroides species in health and disease. Gut Microbes 13(1), 1–20. 10.1080/19490976.2020.1848158. PMC7872030 33535896

[r41] Zheng J, Wittouck S, Salvetti E, Franz CMAP, Harris HMB, Mattarelli P, O’Toole PW, Pot B, Vandamme P, Walter J, Watanabe K, Wuyts S, Felis GE, Gänzle MG and Lebeer S (2020) A taxonomic note on the genus *Lactobacillus*: Description of 23 novel genera, emended description of the genus *Lactobacillus* Beijerinck 1901, and union of *Lactobacillaceae* and *Leuconostocaceae*. International Journal of Systematic and Evolutionary Microbiology 70(4), 2782–2858. 10.1099/ijsem.0.004107. 32293557

[r42] Zúñiga M, Monedero V and Yebra MJ (2018) Utilization of host-derived glycans by intestinal *Lactobacillus* and *Bifidobacterium* species. Frontiers in Microbiology 9. 10.3389/fmicb.2018.01917. PMC6109692 30177920

