# Supplementary material for: Exploring the prebiotic potential of commercial cellobiose: a randomized, controlled trial
Source: Gut Microbiome (Camb). 2026 Jul 27;7:e13. doi: 10.1017/gmb.2026.10032 (PMC13433336; doi:10.1017/gmb.2026.10032)
Supplement: Supplementary file 1 [file S2632289726100322sup001.docx]

**Supplementary Table 1	Mean bacterial populations measured using FISH-flow at V1 and V5 of the intervention phase**

| Intervention | | | | | | | | | |
| --- | --- | --- | --- | --- | --- | --- | --- | --- | --- |
|  | **Cellobiose (*n* = 12)** | | | **OF P95 (*n* = 12)** | | | **Maltodextrin (*n* = 13)** | | |
| Probe | **V1** | **V5** | ***P* (a)** | **V1** | **V5** | ***P* (a)** | **V1** | **V5** | ***P* (a)** |
| Eub I-II-III | 9.39 (0.81) | 9.36 (0.88) | 0.760 | 9.60 (0.81) | 9.59 (0.88) | 0.916 | 9.52 (0.78) | 9.48 (0.85) | 0.634 |

Bacterial groups measured by FISH-flow (Log10 cells/mL) using probes: total bacteria (Eub338 I-II-III) collected at V1and V5 of the intervention phase. Mean and Standard error (SE). **(a)** Significant differences compared with respective V1 and V5 samples are identified with specified *P* values (grey columns) and are result of a general linear model. **Abbreviations:** OF P95 = oligofructose

**Supplementary Table 2	Quantitative Microbiome Profiling (QMP) 16S rRNA data measured at V1 and V5 of the intervention phase**

|  |  | | | **Intervention** | | |  | | |
| --- | --- | --- | --- | --- | --- | --- | --- | --- | --- |
| **Phylum** | **Cellobiose (*n* = 12)** | | | **OF P95 (*n* = 12)** | | | **Maltodextrin (*n* = 13)** | | |
|  | **V1** | **V5** | ***P*** | **V1** | **V5** | ***P*** | **V1** | **V5** | ***P*** |
| **Actinomycetota (Actinobacteria)** | 2.25 x 10^8^ (1.07 x 10^8^) | 3.40 x 10^8^ (1.08 x 10^8^) | 0.327 | 4.22 x 10^8^ (1.07 x 10^8^) | 5.93 x 10^8^ (1.08 x 10^8^) | 0.149 | 3.86 x 10^8^  (1.02 x 10^8^) | 2.87 x 10^8^ (1.03 x 10^8^) | 0.381 |
| **Bacillota (Firmicutes)** | 1.87 x 10^9^ (3.74 x 10^8^) | 1.89 × 10^9^ (4.10 × 10^8^) | 0.973 | 2.78 × 10^9^ (3.74 × 10^8^) | 2.71 × 10^9^ (4.10 × 10^8^) | 0.873 | 2.52 × 10^9^ (3.60 × 10^8^) | 2.29 × 10^9^ (3.94 × 10^8^) | 0.578 |
| **Bacteroidota (Bacteroidetes)** | 8.99 × 10^8^ (2.05 × 10^8^) | 7.48 × 10^8^ (1.45 × 10^8^) | 0.384 | 1.06 × 10^9^ (2.05 × 10^8^) | 9.93 × 10^8^ (1.45 × 10^8^) | 0.675 | 9.32 × 10^8^ (1.97 × 10^8^) | 8.47 × 10^8^ (1.40 × 10^8^) | 0.101 |
| **Euryarchaeota** | 4.60 × 10^6^ (1.19 × 10^7^) | 6.90 × 10^6^ (5.71 × 10^6^) | 0.775 | 1.95 × 10^7^ (1.19 × 10^7^) | 3.19 × 10^6^ (5.71 × 10^6^) | **0.050** | 3.19 × 10^6^ (5.71 × 10^6^) | 1.18 × 10^7^ (5.48 × 10^6^) | 0.182 |
| **Mycoplasmatota (Tenericutes)** | 3.34 × 10^5^ (1.20 × 10^6^) | 4.42 × 10^4^ (7.46 × 10^5^) | 0.547 | 1.13 × 10^5^ (1.20 × 10^6^) | 2.33 × 10^-10^ (7.46 × 10^5^) | 0.814 | 1.92 x 10^6^  (1.16 x 10^6^) | 1.21 x 10^6^ (7.17 x 10^5^) | 0.127 |
| **Pseudomonadota (Proteobacteria)** | 6.92 × 10^7^ (2.39 × 10^7^) | 6.68 ×10^7^ (2.31× 10^7^) | 0.915 | 1.19 × 10^8^ (2.39 × 10^7^) | 6.57 × 10^7^ (2.31 × 10^7^) | **0.021** | 7.95 × 10^7^ (2.29 × 10^7^) | 7.95 × 10^7^ (2.22 × 10^7^) | 1.000 |
| **Thermodesulfobacteriota** | 1.21 × 10^7^ (7.51 × 10^6^) | 1.03 × 10^7^ (4.97 × 10^6^) | 0.678 | 1.24 × 10^7^ (7.51 × 10^6^) | 1.34 × 10^7^ (4.97 × 10^6^) | 0.825 | 2.26 × 10^7^ (7.22 × 10^6^) | 1.58 × 10^7^ (4.77 × 10^6^) | 0.126 |
| **Verrucomicrobiota (Verrucomicrobia*)*** | 5.31 × 10^6^ (8.93 × 10^6^) | 2.07 × 10^7^ (9.82 × 10^6^) | 0.093 | 1.22 × 10^7^ (8.93 × 10^6^) | 1.60 × 10^7^ (9.82 × 10^6^) | 0.673 | 2.62 × 10^7^ (8.58 × 10^6^) | 1.31 × 10⁷ (9.44 × 10^6^) | 0.135 |

## Quantitative microbiome profiling of 16S rRNA gene sequencing data at the phylum level from samples collected at Visit 1 and Visit 5 during the intervention. Values are mean and standard error (SE), expressed as cells per gram of wet faeces. Grey columns mark phyla with significant change from baseline within each intervention arm; P values are from a general linear model. **Abbreviations:** OF P95, oligofructose P95

## **Supplementary Table 3 Quantitative Microbiome Profiling (QMP) 16S rRNA data measured at V1 and V5 of the intervention phase**

|  |  | | | Intervention | | |  | | |
| --- | --- | --- | --- | --- | --- | --- | --- | --- | --- |
| Genus | **Cellobiose (n = 12)** | | | **OF P95 (n = 12)** | | | **Maltodextrin ( n = 13)** | | |
|  | **V1** | **V5** | ***P*** | **V1** | **V5** | ***P*** | **V1** | **V5** | ***P*** |
| *Agathobacter* | 6.22 × 10^7^  (1.89 × 10^7^) | 6.67 × 10^7^  (3.37 × 10^7^) | 0.844 | 1.15 × 10^8^  (2.99 × 10^7^) | 1.17 × 10^8^  (5.04 × 10^7^) | 0.949 | 1.91 × 10^8^  (7.32 × 10^7^) | 1.89 × 10^8^  (6.54 × 10^7^) | 0.973 |
| *Anaerobutyricum* | 5.82 × 10^7^  (1.05 × 10^7^) | 7.18 × 10^7^  (1.60 × 10^7^) | 0.507 | 8.96 × 10^7^  (1.88 × 10^7^) | 7.27 × 10^7^  (1.08 × 10^7^) | 0.416 | 1.30 × 10^8^  (4.17 × 10^7^) | 9.26 × 10^7^  (2.32 × 10^7^) | 0.300 |
| *Anaerostipes* | 5.26 × 10^7^  (1.61 × 10^7^) | 7.41 × 10^7^  (2.46 × 10^7^) | 0.346 | 5.08 × 10^7^  (1.44 × 10^7^) | 8.13 × 10^7^  (2.09 × 10^7^) | 0.089 | 8.85 × 10^7^  (2.30 × 10^7^) | 6.92 × 10^7^  (2.41 × 10^7^) | 0.232 |
| *Bacteroides* | 1.07 × 10^8^  (3.92 × 10^7^) | 1.05 × 10^8^  (3.81 × 10^7^) | 0.942 | 1.45 × 10^8^  (5.21 × 10^7^) | 1.41 × 10^8^  (4.76 × 10^7^) | 0.881 | 1.74 × 10^8^  (7.30 × 10^7^) | 1.31 × 10^8^  (4.27 × 10^7^) | 0.423 |
| *Bifidobacterium* | 1.58 × 10^8^  (4.81 × 10^7^) | 2.46 × 10^8^  (7.26 × 10^7^) | 0.233 | 2.92 × 10^8^  (8.79 × 10^7^) | 4.66 × 10^8^  (1.05 × 10^8^) | 0.049 | 3.00 × 10^8^  (1.00 × 10^8^) | 2.23 × 10^8^  (9.39 × 10^7^) | 0.074 |
| *Blautia* | 2.25 × 10^8^  (4.27 × 10^7^) | 2.37 × 10^8^  (4.78 × 10^7^) | 0.776 | 2.89 × 10^8^  (6.02 × 10^7^) | 2.39 × 10^8^  (4.44 × 10^7^) | 0.473 | 3.67 × 10^8^  (6.71 × 10^7^) | 3.25 × 10^8^  (5.73 × 10^7^) | 0.537 |
| *Collinsella* | 5.51 × 10^7^  (1.32 × 10^7^) | 7.61 × 10^7^  (1.69 × 10^7^) | 0.280 | 1.08 × 10^8^  (4.71 × 10^7^) | 9.98 × 10^7^  (2.67 × 10^7^) | 0.867 | 6.58 × 10^7^  (2.34 × 10^7^) | 5.18 × 10^7^  (1.55 × 10^7^) | 0.562 |
| Genus (continued) | **Cellobiose (n = 12)** | | | **OF P95 (n = 12)** | | | **Maltodextrin ( n = 13)** | | |
|  | **V1** | **V5** | ***P*** | **V1** | **V5** | ***P*** | **V1** | **V5** | ***P*** |
| *Coprococcus* | 4.90 × 10^7^  (1.63 × 10^7^) | 6.18 × 10^7^  (1.91 × 10^7^) | 0.532 | 1.03 × 10^8^  (2.20 × 10^7^) | 9.90 × 10^7^  (3.72 × 10^7^) | 0.901 | 5.48 × 10^7^  (1.05 × 10^7^) | 7.96 × 10^7^  (1.93 × 10^7^) | 0.134 |
| *Dialister* | 5.52 × 10^7^  (1.38 × 10^7^) | 4.72 × 10^07  (1.52 × 10^07) | 0.537 | 1.26 × 10^8^  (4.21 × 10^7^) | 1.02 × 10^8^  (2.12 × 10^7^) | 0.539 | 5.11 × 10^7^  (1.90 × 10^7^) | 5.49 × 10^7^  (1.92 × 10^7^) | 0.851 |
| *Dorea* | 5.60 × 10^7^  (9.96 × 10^6^) | 4.65 × 10^07  (8.20 × 10^06) | 0.397 | 9.02 × 10^7^  (2.54 × 10^7^) | 6.75 × 10^7^  (1.25 × 10^7^) | 0.393 | 4.94 × 10^7^  (1.04 × 10^7^) | 5.16 × 10^7^  (1.21 × 10^7^) | 0.881 |
| *Faecalibacterium* | 2.50 × 10^8^  (4.70 × 10^7^) | 2.50 × 10^08  (5.21 × 10^07) | 0.993 | 2.92 × 10^8^  (5.05 × 10^7^) | 2.90 × 10^8^  (6.31 × 10^7^) | 0.966 | 3.73 × 10^8^  (1.04 × 10^8^) | 3.53 × 10^8^  (1.07 × 10^8^) | 0.688 |
| *Fusicatenibacter* | 7.30 × 10^7^  (2.19 × 10^7^) | 9.75 × 10^07  (2.73 × 10^07) | 0.224 | 7.48 × 10^7^  (1.91 × 10^7^) | 7.57 × 10^7^  (2.11 × 10^7^) | 0.944 | 1.13 × 10^8^  (3.94 × 10^7^) | 1.06 × 10^8^  (3.68 × 10^7^) | 0.739 |
| *Gemmiger* | 3.98 × 10^07  (1.38 × 10^07) | 5.09 × 10^07  (1.71 × 10^07) | 0.423 | 7.94 × 10^7^  (1.81 × 10^7^) | 5.83 × 10^7^  (1.56 × 10^7^) | 0.205 | 5.67 × 10^7^  (1.47 × 10^7^) | 6.05 × 10^7^  (1.69 × 10^7^) | 0.749 |
| *Lientehia* | 2.32 × 10^07  (6.81 × 10^06) | 2.20 × 10^07  (4.89 × 10^06) | 0.875 | 2.38 × 10^7^  (5.45 × 10^6^) | 3.37 × 10^7^  (1.05 × 10^7^) | 0.342 | 3.96 × 10^7^  (1.53 × 10^7^) | 3.05 × 10^7^  (1.02 × 10^7^) | 0.306 |
| *Mediterraneibacter* | 4.83 × 10^07  (1.21 × 10^07) | 2.57 × 10^07  (5.33 × 10^06) | 0.090 | 5.71 × 10^7^  (1.18 × 10^7^) | 3.09 × 10^7^  (6.36 × 10^6^) | 0.051 | 6.39 × 10^7^  (1.93 × 10^7^) | 5.61 × 10^7^  (1.04 × 10^7^) | 0.710 |
| Genus (continued) | **Cellobiose (n = 12)** | | | **OF P95 (n = 12)** | | | **Maltodextrin ( n = 13)** | | |
|  | **V1** | **V5** | ***P*** | **V1** | **V5** | ***P*** | **V1** | **V5** | ***P*** |
| *Parabacteroides* | 2.86 × 10^7^  (8.50 × 10^6^) | 2.79 × 10^7^  (8.19 × 10^6^) | 0.912 | 3.00 × 10^7^  (6.12 × 10^6^) | 3.76 × 10^7^  (1.07 × 10^7^) | 0.302 | 5.69 × 10^7^  (2.06 × 10^7^) | 5.63 × 10^7^  (1.43 × 10^7^) | 0.960 |
| *Phocaeicola* | 2.03 × 10^8^  (8.99 × 10^7^) | 1.54 × 10^8^  (5.69 × 10^7^) | 0.324 | 2.21 × 10^8^  (6.77 × 10^7^) | 2.41 × 10^8^  (7.07 × 10^7^) | 0.627 | 1.99 × 10^8^  (3.98 × 10^7^) | 2.37 × 10^8^  (5.17 × 10^7^) | 0.176 |
| *Romboutsia* | 3.82 × 10^7^  (1.42 × 10^7^) | 3.06 × 10^7^  (1.22 × 10^7^) | 0.573 | 4.49 × 10^7^  (9.93 × 10^6^) | 5.81 × 10^7^  (2.56 × 10^7^) | 0.638 | 4.04 × 10^7^  (8.57 × 10^6^) | 3.24 × 10^7^  (7.29 × 10^6^) | 0.441 |
| *Roseburia* | 8.81 × 10^7^  (2.01 × 10^7^) | 7.65 × 10^7^  (1.97 × 10^7^) | 0.630 | 1.64 × 10^8^  (4.75 × 10^7^) | 1.41 × 10^8^  (5.52 × 10^7^) | 0.658 | 9.69 × 10^7^  (3.05 × 10^7^) | 5.79 × 10^7^  (1.43 × 10^7^) | 0.220 |
| *Ruminococcus* | 5.68 × 10^7^  (2.15 × 10^7^) | 7.92 × 10^7^  (4.13 × 10^7^) | 0.545 | 1.12 × 10^8^  (3.44 × 10^7^) | 4.83 × 10^7^  (1.09 × 10^7^) | 0.097 | 1.21 × 10^8^  (3.44 × 10^7^) | 1.16 × 10^8^  (4.70 × 10^7^) | 0.867 |
| *Segatella* | 4.46 × 10^8^  (1.72 × 10^8^) | 3.69 × 10^8^  (1.41 × 10^8^) | 0.498 | 5.24 × 10^8^  (2.08 × 10^8^) | 4.08 × 10^8^  (1.87 × 10^8^) | 0.406 | 3.63 × 10^8^  (2.12 × 10^8^) | 2.99 × 10^8^  (1.26 × 10^8^) | 0.687 |
| *Streptococcus* | 1.94 × 10^7^  (7.28 × 10^6^) | 1.87 × 10^7^  (6.69 × 10^6^) | 0.944 | 3.22 × 10^7^  (1.48 × 10^7^) | 2.40 × 10^7^  (1.27 × 10^7^) | 0.136 | 2.68 × 10^7^  (9.59 × 10^6^) | 4.34 × 10^7^  (2.23 × 10^7^) | 0.293 |
| *Sutterella* | 5.25 × 10^7^  (1.19 × 10^7^) | 4.10 × 10^7^  (1.09 × 10^7^) | 0.363 | 4.67 × 10^7^  (2.07 × 10^7^) | 3.29 × 10^7^  (1.22 × 10^7^) | 0.474 | 2.90 × 10^7^  (9.55 × 10^6^) | 3.67 × 10^7^  (1.82 × 10^7^) | 0.638 |
| *Vescimonas* | 1.03 × 10^7^  (3.24 × 10^6^) | 1.28 × 10^7^  (6.16 × 10^6^) | 0.696 | 2.86 × 10^7^  (8.59 × 10^6^) | 2.25 × 10^7^  (8.80 × 10^6^) | 0.342 | 4.98 × 10^7^  (2.48 × 10^7^) | 2.86 × 10^7^  (7.56 × 10^6^) | 0.331 |

Quantitative Microbiome Profiling 16S rRNA sequencing data (genus level) from samples collected at V1 and V5 of the intervention phase. Mean and Standard error (SE). Significant differences compared with respective D0 and D28 samples are identified with specified *P* values (grey columns) and are result of a general linear model. **Abbreviations:** OF P95= oligofructose P95

## **Supplementary Table 4 Bacterial genus-genus correlation matrix**

genus-genus correlation by group: cellobiose

|  | ***Agathobacter*** | ***Anaerobutyricum*** | ***Anaerostipes*** | ***Bacteroides*** | ***Bifidobacterium*** | ***Blautia*** | ***Collinsella*** | ***Coprococcus*** | ***Dialister*** | ***Dorea*** | ***Faecalibacterium*** | ***Fusicatenibacter*** | ***Gemmiger*** | ***Lientehia*** | ***NA*** | ***Mediterraneibacter*** | ***Parabacteroides*** | ***Phocaeicola*** | ***Romboutsia*** | ***Roseburia*** | ***Ruminococcus*** | ***Segatella*** | ***Streptococcus*** | ***Sutterella*** | ***Vescimonas*** |
| --- | --- | --- | --- | --- | --- | --- | --- | --- | --- | --- | --- | --- | --- | --- | --- | --- | --- | --- | --- | --- | --- | --- | --- | --- | --- |
| ***Agathobacter*** | 1.000 | 0.235 | 0.165 | 0.123 | 0.347 | 0.228 | -0.011 | 0.095 | 0.194 | 0.193 | 0.308 | 0.056 | 0.354 | -0.168 | 0.039 | -0.053 | 0.067 | -0.165 | 0.105 | -0.095 | 0.435 | 0.442 | 0.119 | 0.378 | -0.142 |
|  | 1.000 | 0.530 | 0.665 | 0.749 | 0.347 | 0.544 | 0.974 | 0.797 | 0.615 | 0.616 | 0.407 | 0.878 | 0.338 | 0.664 | 0.915 | 0.883 | 0.860 | 0.665 | 0.785 | 0.797 | 0.235 | 0.229 | 0.755 | 0.304 | 0.712 |
| ***Anaerobutyricum*** | 0.235 | 1.000 | 0.832 | 0.622 | 0.881 | 0.797 | 0.385 | 0.748 | 0.416 | 0.839 | 0.699 | 0.860 | 0.825 | 0.559 | 0.329 | 0.734 | 0.168 | 0.469 | 0.441 | 0.448 | 0.515 | 0.498 | 0.776 | 0.343 | 0.466 |
|  | 0.530 | 1.000 | **0.005** | **0.079** | **0.002** | **0.010** | 0.296 | **0.021** | 0.255 | **0.005** | **0.036** | **0.003** | **0.006** | 0.121 | 0.373 | **0.025** | 0.664 | 0.210 | 0.229 | 0.225 | 0.162 | 0.180 | **0.015** | 0.352 | 0.213 |
| ***Anaerostipes*** | 0.165 | 0.832 | 1.000 | 0.825 | 0.839 | 0.979 | 0.413 | 0.916 | 0.549 | 0.881 | 0.923 | 0.923 | 0.937 | 0.860 | 0.455 | 0.916 | 0.322 | 0.497 | 0.098 | 0.483 | 0.680 | 0.673 | 0.790 | 0.657 | 0.235 |
|  | 0.665 | **0.005** | 1.000 | **0.006** | **0.005** | **9.27×10^-6^** | 0.255 | **0.001** | 0.130 | **0.002** | **0.001** | **0.001** | **3.00×10^-4^** | **0.003** | 0.220 | **0.001** | 0.385 | 0.180 | 0.797 | 0.193 | **0.047** | **0.050** | **0.011** | 0.057 | 0.530 |
| ***Bacteroides*** | 0.123 | 0.622 | 0.825 | 1.000 | 0.720 | 0.874 | 0.699 | 0.895 | 0.620 | 0.797 | 0.867 | 0.804 | 0.853 | 0.804 | 0.538 | 0.811 | 0.769 | 0.490 | 0.217 | 0.273 | 0.578 | 0.634 | 0.559 | 0.608 | 0.375 |
|  | 0.749 | 0.079 | **0.006** | 1.000 | **0.029** | **0.002** | 0.036 | 0.001 | 0.080 | 0.010 | 0.003 | 0.009 | 0.003 | 0.009 | 0.138 | 0.008 | 0.016 | 0.185 | 0.567 | 0.460 | 0.107 | 0.071 | 0.121 | 0.085 | 0.308 |
| ***Bifidobacterium*** | 0.347 | 0.881 | 0.839 | 0.720 | 1.000 | 0.825 | 0.594 | 0.839 | 0.585 | 0.874 | 0.853 | 0.860 | 0.902 | 0.566 | 0.469 | 0.762 | 0.343 | 0.448 | 0.441 | 0.427 | 0.651 | 0.701 | 0.671 | 0.517 | 0.515 |
|  | 0.347 | **0.002** | **0.005** | **0.029** | 1.000 | **0.006** | 0.095 | **0.005** | 0.102 | **0.002** | **0.003** | **0.003** | **0.001** | 0.117 | 0.210 | **0.017** | 0.352 | 0.225 | 0.229 | 0.242 | 0.061 | **0.036** | **0.050** | 0.160 | 0.162 |
| ***Blautia*** | 0.228 | 0.797 | 0.979 | 0.874 | 0.825 | 1.000 | 0.441 | 0.944 | 0.599 | 0.846 | 0.923 | 0.895 | 0.951 | 0.860 | 0.413 | 0.909 | 0.413 | 0.427 | 0.105 | 0.455 | 0.760 | 0.680 | 0.804 | 0.720 | 0.235 |
|  | 0.544 | **0.010** | **9.27×10^-6^** | **0.002** | **0.006** | 1.000 | 0.229 | **2.95 × 10⁻⁴** | 0.092 | **0.004** | **0.001** | **0.001** | **2.95 × 10⁻⁴** | **0.003** | 0.255 | **0.001** | 0.255 | 0.242 | 0.785 | 0.220 | **0.018** | **0.047** | **0.009** | **0.029** | 0.530 |
| ***Collinsella*** | -0.011 | 0.385 | 0.413 | 0.699 | 0.594 | 0.441 | 1.000 | 0.615 | 0.451 | 0.657 | 0.608 | 0.538 | 0.531 | 0.371 | 0.783 | 0.566 | 0.825 | 0.643 | 0.671 | 0.399 | 0.280 | 0.434 | 0.238 | 0.287 | 0.774 |
|  | 0.974 | 0.296 | 0.255 | **0.036** | 0.095 | 0.229 | 1.000 | 0.081 | 0.224 | 0.057 | 0.085 | 0.138 | 0.144 | 0.314 | **0.013** | 0.117 | **0.006** | 0.064 | **0.050** | 0.276 | 0.450 | 0.235 | 0.529 | 0.440 | **0.015** |
| ***Coprococcus*** | 0.095 | 0.748 | 0.916 | 0.895 | 0.839 | 0.944 | 0.615 | 1.000 | 0.746 | 0.853 | 0.895 | 0.888 | 0.937 | 0.832 | 0.531 | 0.895 | 0.517 | 0.455 | 0.287 | 0.545 | 0.729 | 0.715 | 0.769 | 0.657 | 0.378 |
|  | 0.797 | **0.021** | **0.001** | **0.001** | **0.005** | **3.00 × 10⁻⁴** | 0.081 | 1.000 | **0.021** | **0.003** | **0.001** | **0.002** | **2.95 × 10⁻⁴** | **0.005** | 0.144 | **0.001** | 0.160 | 0.220 | 0.440 | 0.132 | **0.026** | **0.031** | **0.016** | 0.057 | 0.304 |
| ***Dialister*** | 0.194 | 0.416 | 0.549 | 0.620 | 0.585 | 0.599 | 0.451 | 0.746 | 1.000 | 0.458 | 0.620 | 0.500 | 0.732 | 0.430 | 0.338 | 0.479 | 0.472 | 0.070 | 0.303 | 0.282 | 0.487 | 0.763 | 0.352 | 0.352 | 0.250 |
|  | 0.615 | 0.255 | 0.130 | 0.080 | 0.102 | 0.092 | 0.224 | **0.021** | 1.000 | 0.219 | 0.080 | 0.180 | **0.025** | 0.240 | 0.359 | 0.198 | 0.207 | 0.853 | 0.416 | 0.448 | 0.188 | **0.017** | 0.338 | 0.338 | 0.505 |
| ***Dorea*** | 0.193 | 0.839 | 0.881 | 0.797 | 0.874 | 0.846 | 0.657 | 0.853 | 0.458 | 1.000 | 0.867 | 0.937 | 0.874 | 0.727 | 0.706 | 0.811 | 0.420 | 0.727 | 0.441 | 0.622 | 0.585 | 0.644 | 0.734 | 0.608 | 0.413 |
|  | 0.616 | **0.005** | **0.002** | **0.010** | **0.002** | **0.004** | 0.057 | **0.003** | 0.219 | 1.000 | **0.003** | **3.00 × 10⁻⁴** | **0.002** | **0.026** | **0.035** | **0.008** | 0.249 | 0.026 | 0.229 | 0.079 | 0.102 | 0.064 | **0.025** | 0.085 | 0.255 |
| ***Faecalibacterium*** | 0.308 | 0.699 | 0.923 | 0.867 | 0.853 | 0.923 | 0.608 | 0.895 | 0.620 | 0.867 | 1.000 | 0.846 | 0.944 | 0.769 | 0.615 | 0.874 | 0.538 | 0.545 | 0.189 | 0.434 | 0.701 | 0.758 | 0.601 | 0.699 | 0.336 |
|  | 0.407 | **0.036** | **0.001** | **0.003** | **0.003** | **0.001** | 0.085 | **0.001** | 0.080 | **0.003** | 1.000 | **0.004** | **2.95 × 10⁻⁴** | **0.016** | 0.081 | **0.002** | 0.138 | 0.132 | 0.623 | 0.235 | **0.036** | **0.018** | 0.090 | **0.036** | 0.361 |
| ***Fusicatenibacter*** | 0.056 | 0.860 | 0.923 | 0.804 | 0.860 | 0.895 | 0.538 | 0.888 | 0.500 | 0.937 | 0.846 | 1.000 | 0.895 | 0.846 | 0.490 | 0.853 | 0.357 | 0.678 | 0.315 | 0.580 | 0.606 | 0.594 | 0.755 | 0.615 | 0.368 |
|  | 0.878 | **0.003** | **0.001** | **0.009** | **0.003** | **0.001** | 0.138 | **0.002** | 0.180 | 0.000 | 0.004 | 1.000 | 0.001 | 0.004 | 0.185 | 0.003 | 0.336 | 0.047 | 0.396 | 0.105 | 0.087 | 0.095 | 0.019 | 0.081 | 0.318 |
| ***Gemmiger*** | 0.354 | 0.825 | 0.937 | 0.853 | 0.902 | 0.951 | 0.531 | 0.937 | 0.732 | 0.874 | 0.944 | 0.895 | 1.000 | 0.748 | 0.490 | 0.832 | 0.462 | 0.462 | 0.294 | 0.462 | 0.736 | 0.783 | 0.706 | 0.657 | 0.329 |
|  | 0.338 | **0.006** | **3.00 × 10⁻⁴** | **0.003** | **0.001** | **2.95 × 10⁻⁴** | 0.144 | **3.00 × 10⁻⁴** | **0.025** | **0.002** | **2.95 × 10⁻⁴** | **0.001** | 1.000 | **0.021** | 0.185 | **0.005** | 0.216 | 0.216 | 0.430 | 0.216 | **0.025** | **0.013** | **0.035** | 0.057 | 0.373 |
| ***Lientehia*** | -0.168 | 0.559 | 0.860 | 0.804 | 0.566 | 0.860 | 0.371 | 0.832 | 0.430 | 0.727 | 0.769 | 0.846 | 0.748 | 1.000 | 0.392 | 0.825 | 0.385 | 0.552 | -0.056 | 0.448 | 0.630 | 0.409 | 0.643 | 0.657 | 0.095 |
|  | 0.664 | 0.121 | **0.003** | **0.009** | 0.117 | 0.003 | 0.314 | **0.005** | 0.240 | **0.026** | **0.016** | **0.004** | **0.021** | 1.000 | 0.286 | **0.006** | 0.296 | 0.128 | 0.878 | 0.225 | 0.073 | 0.259 | 0.064 | 0.057 | 0.797 |
| ***NA*** | 0.039 | 0.329 | 0.455 | 0.538 | 0.469 | 0.413 | 0.783 | 0.531 | 0.338 | 0.706 | 0.615 | 0.490 | 0.490 | 0.392 | 1.000 | 0.510 | 0.552 | 0.741 | 0.545 | 0.587 | 0.287 | 0.427 | 0.273 | 0.315 | 0.455 |
|  | 0.915 | 0.373 | 0.220 | 0.138 | 0.210 | 0.255 | **0.013** | 0.144 | 0.359 | **0.035** | 0.081 | 0.185 | 0.185 | 0.286 | 1.000 | 0.167 | 0.128 | **0.023** | 0.132 | 0.101 | 0.440 | 0.242 | 0.460 | 0.396 | 0.220 |
| ***Mediterraneibacter*** | -0.053 | 0.734 | 0.916 | 0.811 | 0.762 | 0.909 | 0.566 | 0.895 | 0.479 | 0.811 | 0.874 | 0.853 | 0.832 | 0.825 | 0.510 | 1.000 | 0.441 | 0.559 | 0.161 | 0.559 | 0.581 | 0.566 | 0.755 | 0.587 | 0.459 |
|  | 0.883 | **0.025** | **0.001** | **0.008** | **0.017** | **0.001** | 0.117 | **0.001** | 0.198 | **0.008** | **0.002** | **0.003** | **0.005** | **0.006** | 0.167 | 1.000 | 0.229 | 0.121 | 0.671 | 0.121 | 0.105 | 0.117 | **0.019** | 0.101 | 0.219 |
| ***Parabacteroides*** | 0.067 | 0.168 | 0.322 | 0.769 | 0.343 | 0.413 | 0.825 | 0.517 | 0.472 | 0.420 | 0.538 | 0.357 | 0.462 | 0.385 | 0.552 | 0.441 | 1.000 | 0.420 | 0.378 | 0.035 | 0.238 | 0.356 | 0.035 | 0.273 | 0.532 |
|  | 0.860 | 0.664 | 0.385 | **0.016** | 0.352 | 0.255 | **0.006** | 0.160 | 0.207 | 0.249 | 0.138 | 0.336 | 0.216 | 0.296 | 0.128 | 0.229 | 1.000 | 0.249 | 0.304 | 0.917 | 0.529 | 0.336 | 0.917 | 0.460 | 0.144 |
| ***Phocaeicola*** | -0.165 | 0.469 | 0.497 | 0.490 | 0.448 | 0.427 | 0.643 | 0.455 | 0.070 | 0.727 | 0.545 | 0.678 | 0.462 | 0.552 | 0.741 | 0.559 | 0.420 | 1.000 | 0.497 | 0.615 | 0.196 | 0.174 | 0.294 | 0.294 | 0.490 |
|  | 0.665 | 0.210 | 0.180 | 0.185 | 0.225 | 0.242 | 0.064 | 0.220 | 0.853 | **0.026** | 0.132 | **0.047** | 0.216 | 0.128 | **0.023** | 0.121 | 0.249 | 1.000 | 0.180 | 0.081 | 0.613 | 0.653 | 0.430 | 0.430 | 0.185 |
| ***Romboutsia*** | 0.105 | 0.441 | 0.098 | 0.217 | 0.441 | 0.105 | 0.671 | 0.287 | 0.303 | 0.441 | 0.189 | 0.315 | 0.294 | -0.056 | 0.545 | 0.161 | 0.378 | 0.497 | 1.000 | 0.448 | 0.137 | 0.125 | 0.175 | -0.126 | 0.764 |
|  | 0.785 | 0.229 | 0.797 | 0.567 | 0.229 | 0.785 | **0.050** | 0.440 | 0.416 | 0.229 | 0.623 | 0.396 | 0.430 | 0.878 | 0.132 | 0.671 | 0.304 | 0.180 | 1.000 | 0.225 | 0.723 | 0.747 | 0.653 | 0.746 | 0.017 |
| ***Roseburia*** | -0.095 | 0.448 | 0.483 | 0.273 | 0.427 | 0.455 | 0.399 | 0.545 | 0.282 | 0.622 | 0.434 | 0.580 | 0.462 | 0.448 | 0.587 | 0.559 | 0.035 | 0.615 | 0.448 | 1.000 | 0.424 | 0.367 | 0.678 | 0.497 | 0.266 |
|  | 0.797 | 0.225 | 0.193 | 0.460 | 0.242 | 0.220 | 0.276 | 0.132 | 0.448 | 0.079 | 0.235 | 0.105 | 0.216 | 0.225 | 0.101 | 0.121 | 0.917 | 0.081 | 0.225 | 1.000 | 0.245 | 0.319 | **0.047** | 0.180 | 0.472 |
| ***Ruminococcus*** | 0.435 | 0.515 | 0.680 | 0.578 | 0.651 | 0.760 | 0.280 | 0.729 | 0.487 | 0.585 | 0.701 | 0.606 | 0.736 | 0.630 | 0.287 | 0.581 | 0.238 | 0.196 | 0.137 | 0.424 | 1.000 | 0.437 | 0.648 | 0.746 | 0.065 |
|  | 0.235 | 0.162 | **0.047** | 0.107 | 0.061 | **0.018** | 0.450 | **0.026** | 0.188 | 0.102 | **0.036** | 0.087 | **0.025** | 0.073 | 0.440 | 0.105 | 0.529 | 0.613 | 0.723 | 0.245 | 1.000 | 0.234 | 0.063 | **0.021** | 0.861 |
| ***Segatella*** | 0.442 | 0.498 | 0.673 | 0.634 | 0.701 | 0.680 | 0.434 | 0.715 | 0.763 | 0.644 | 0.758 | 0.594 | 0.783 | 0.409 | 0.427 | 0.566 | 0.356 | 0.174 | 0.125 | 0.367 | 0.437 | 1.000 | 0.498 | 0.644 | 0.098 |
|  | 0.229 | 0.180 | **0.050** | 0.071 | **0.036** | **0.047** | 0.235 | **0.031** | **0.017** | 0.064 | **0.018** | 0.095 | **0.013** | 0.259 | 0.242 | 0.117 | 0.336 | 0.653 | 0.747 | 0.319 | 0.234 | 1.000 | 0.180 | 0.064 | 0.797 |
| ***Streptococcus*** | 0.119 | 0.776 | 0.790 | 0.559 | 0.671 | 0.804 | 0.238 | 0.769 | 0.352 | 0.734 | 0.601 | 0.755 | 0.706 | 0.643 | 0.273 | 0.755 | 0.035 | 0.294 | 0.175 | 0.678 | 0.648 | 0.498 | 1.000 | 0.636 | 0.151 |
|  | 0.755 | **0.015** | **0.011** | 0.121 | **0.050** | **0.009** | 0.529 | **0.016** | 0.338 | **0.025** | **0.090** | **0.019** | **0.035** | **0.064** | 0.460 | **0.019** | 0.917 | 0.430 | 0.653 | **0.047** | 0.063 | 0.180 | 1.000 | 0.069 | 0.694 |
| ***Sutterella*** | 0.378 | 0.343 | 0.657 | 0.608 | 0.517 | 0.720 | 0.287 | 0.657 | 0.352 | 0.608 | 0.699 | 0.615 | 0.657 | 0.657 | 0.315 | 0.587 | 0.273 | 0.294 | -0.126 | 0.497 | 0.746 | 0.644 | 0.636 | 1.000 | -0.165 |
|  | 0.304 | 0.352 | 0.057 | 0.085 | 0.160 | **0.029** | 0.440 | 0.057 | 0.338 | 0.085 | **0.036** | 0.081 | 0.057 | 0.057 | 0.396 | 0.101 | 0.460 | 0.430 | 0.746 | 0.180 | **0.021** | 0.064 | 0.069 | 1.000 | 0.665 |
| ***Vescimonas*** | -0.142 | 0.466 | 0.235 | 0.375 | 0.515 | 0.235 | 0.774 | 0.378 | 0.250 | 0.413 | 0.336 | 0.368 | 0.329 | 0.095 | 0.455 | 0.459 | 0.532 | 0.490 | 0.764 | 0.266 | 0.065 | 0.098 | 0.151 | -0.165 | 1.000 |
|  | 0.712 | 0.213 | 0.530 | 0.308 | 0.162 | 0.530 | **0.015** | 0.304 | 0.505 | 0.255 | 0.361 | 0.318 | 0.373 | 0.797 | 0.220 | 0.219 | 0.144 | 0.185 | **0.017** | 0.472 | 0.861 | 0.797 | 0.694 | 0.665 | 1.000 |

Bacterial genus-genus interactions from the entire cohort. Pairwise correlations between genus using log_2_ (fold change) data were calculated using a Spearman’s rank correlation (two sided adjusted for using BH-FDR). The correlations ranged from -1 to 1 (negative to positive). Red text indicates adjusted *P* (*Q*) values ≤ 0.05 (grey rows).

Genus-genus correlation by group: OF P95

|  | ***Agathobacter*** | ***Anaerobutyricum*** | ***Anaerostipes*** | ***Bacteroides*** | ***Bifidobacterium*** | ***Blautia*** | ***Collinsella*** | ***Coprococcus*** | ***Dialister*** | ***Dorea*** | ***Faecalibacterium*** | ***Fusicatenibacter*** | ***Gemmiger*** | ***Lientehia*** | ***NA*** | ***Mediterraneibacter*** | ***Parabacteroides*** | ***Phocaeicola*** | ***Romboutsia*** | ***Roseburia*** | ***Ruminococcus*** | ***Segatella*** | ***Streptococcus*** | ***Sutterella*** | ***Vescimonas*** |
| --- | --- | --- | --- | --- | --- | --- | --- | --- | --- | --- | --- | --- | --- | --- | --- | --- | --- | --- | --- | --- | --- | --- | --- | --- | --- |
| ***Agathobacter*** | 1.000 | 0.308 | 0.161 | 0.497 | 0.399 | 0.441 | 0.263 | 0.231 | 0.413 | 0.420 | 0.462 | 0.643 | 0.385 | 0.420 | 0.476 | 0.189 | 0.524 | 0.483 | 0.217 | 0.315 | 0.483 | 0.593 | 0.741 | 0.776 | -0.098 |
|  | 1.000 | 0.496 | 0.744 | 0.236 | 0.362 | 0.306 | 0.569 | 0.627 | 0.338 | 0.329 | 0.281 | 0.113 | 0.379 | 0.329 | 0.263 | 0.699 | 0.209 | 0.251 | 0.653 | 0.488 | 0.251 | 0.138 | **0.050** | **0.036** | 0.841 |
| ***Anaerobutyricum*** | 0.308 | 1.000 | 0.636 | 0.566 | 0.804 | 0.909 | 0.876 | 0.615 | 0.790 | 0.622 | 0.231 | 0.741 | 0.860 | 0.280 | 0.490 | 0.245 | 0.594 | 0.497 | 0.573 | -0.175 | 0.629 | -0.015 | -0.021 | -0.028 | 0.573 |
|  | 0.496 | 1.000 | 0.117 | 0.160 | **0.027** | **0.006** | **0.009** | 0.126 | **0.033** | 0.118 | 0.627 | **0.050** | **0.013** | 0.536 | 0.243 | 0.602 | 0.138 | 0.236 | 0.159 | 0.727 | 0.118 | 0.966 | 0.958 | 0.950 | 0.159 |
| ***Anaerostipes*** | 0.161 | 0.636 | 1.000 | 0.420 | 0.713 | 0.678 | 0.595 | -0.042 | 0.497 | 0.308 | -0.168 | 0.601 | 0.343 | -0.070 | 0.203 | -0.098 | 0.175 | -0.021 | 0.385 | 0.196 | 0.594 | 0.317 | 0.161 | -0.014 | 0.084 |
|  | 0.744 | 0.117 | 1.000 | 0.329 | 0.063 | 0.084 | 0.138 | 0.924 | 0.236 | 0.496 | 0.737 | 0.136 | 0.447 | 0.876 | 0.682 | 0.841 | 0.727 | 0.958 | 0.379 | 0.686 | 0.138 | 0.488 | 0.744 | 0.966 | 0.855 |
| ***Bacteroides*** | 0.497 | 0.566 | 0.420 | 1.000 | 0.748 | 0.692 | 0.651 | 0.566 | 0.594 | 0.650 | 0.238 | 0.664 | 0.664 | 0.503 | 0.741 | 0.448 | 0.566 | 0.650 | 0.629 | -0.056 | 0.818 | 0.060 | 0.399 | 0.455 | 0.455 |
|  | 0.236 | 0.160 | 0.329 | 1.000 | **0.050** | 0.077 | 0.105 | 0.160 | 0.138 | 0.105 | 0.615 | 0.097 | 0.097 | 0.234 | **0.050** | 0.299 | 0.160 | 0.105 | 0.118 | 0.896 | **0.020** | 0.892 | 0.362 | 0.287 | 0.287 |
| ***Bifidobacterium*** | 0.399 | 0.804 | 0.713 | 0.748 | 1.000 | 0.923 | 0.851 | 0.490 | 0.748 | 0.552 | 0.105 | 0.692 | 0.727 | 0.385 | 0.490 | 0.105 | 0.350 | 0.329 | 0.469 | -0.147 | 0.839 | 0.131 | 0.133 | 0.196 | 0.462 |
|  | 0.362 | 0.027 | 0.063 | **0.050** | 1.000 | **0.006** | **0.015** | 0.243 | **0.050** | 0.175 | 0.828 | 0.077 | 0.060 | 0.379 | 0.243 | 0.828 | 0.440 | 0.469 | 0.273 | 0.769 | **0.016** | 0.797 | 0.794 | 0.686 | 0.281 |
| ***Blautia*** | 0.441 | 0.909 | 0.678 | 0.692 | 0.923 | 1.000 | 0.858 | 0.650 | 0.888 | 0.734 | 0.308 | 0.741 | 0.846 | 0.392 | 0.657 | 0.287 | 0.608 | 0.483 | 0.552 | -0.084 | 0.783 | 0.116 | 0.196 | 0.196 | 0.531 |
|  | 0.306 | **0.006** | 0.084 | 0.077 | **0.006** | 1.000 | **0.013** | 0.105 | **0.009** | 0.055 | 0.496 | **0.050** | **0.016** | 0.369 | 0.103 | 0.528 | 0.129 | 0.251 | 0.175 | 0.855 | **0.034** | 0.819 | 0.686 | 0.686 | 0.200 |
| ***Collinsella*** | 0.263 | 0.876 | 0.595 | 0.651 | 0.851 | 0.858 | 1.000 | 0.606 | 0.788 | 0.578 | 0.088 | 0.627 | 0.879 | 0.427 | 0.473 | 0.119 | 0.497 | 0.354 | 0.578 | -0.042 | 0.715 | -0.110 | -0.074 | 0.109 | 0.630 |
|  | 0.569 | **0.009** | 0.138 | 0.105 | **0.015** | **0.013** | 1.000 | 0.131 | **0.033** | 0.157 | 0.855 | 0.118 | **0.009** | 0.327 | 0.266 | 0.813 | 0.236 | 0.437 | 0.157 | 0.924 | 0.063 | 0.827 | 0.873 | 0.828 | 0.118 |
| ***Coprococcus*** | 0.231 | 0.615 | -0.042 | 0.566 | 0.490 | 0.650 | 0.606 | 1.000 | 0.678 | 0.713 | 0.622 | 0.371 | 0.825 | 0.608 | 0.741 | 0.545 | 0.678 | 0.692 | 0.510 | -0.308 | 0.441 | -0.422 | 0.014 | 0.126 | 0.776 |
|  | 0.627 | 0.126 | 0.924 | 0.160 | 0.243 | 0.105 | 0.131 | 1.000 | 0.084 | 0.063 | 0.118 | 0.404 | **0.019** | 0.129 | **0.050** | 0.180 | 0.084 | 0.077 | 0.225 | 0.496 | 0.306 | 0.329 | 0.966 | 0.804 | **0.036** |
| ***Dialister*** | 0.413 | 0.790 | 0.497 | 0.594 | 0.748 | 0.888 | 0.788 | 0.678 | 1.000 | 0.713 | 0.287 | 0.524 | 0.839 | 0.336 | 0.622 | 0.371 | 0.762 | 0.420 | 0.336 | 0.112 | 0.818 | 0.127 | 0.084 | 0.287 | 0.685 |
|  | 0.338 | **0.033** | 0.236 | 0.138 | **0.050** | **0.009** | **0.033** | 0.084 | 1.000 | 0.063 | 0.528 | 0.209 | **0.016** | 0.454 | 0.118 | 0.404 | **0.046** | 0.329 | 0.454 | 0.826 | **0.020** | 0.804 | 0.855 | 0.528 | 0.082 |
| ***Dorea*** | 0.420 | 0.622 | 0.308 | 0.650 | 0.552 | 0.734 | 0.578 | 0.713 | 0.713 | 1.000 | 0.392 | 0.608 | 0.755 | 0.224 | 0.895 | 0.692 | 0.720 | 0.790 | 0.517 | -0.021 | 0.497 | -0.037 | 0.336 | 0.455 | 0.364 |
|  | 0.329 | 0.118 | 0.496 | 0.105 | 0.175 | 0.055 | 0.157 | 0.063 | 0.063 | 1.000 | 0.369 | 0.129 | 0.048 | 0.640 | **0.008** | 0.077 | 0.063 | **0.033** | 0.218 | 0.958 | 0.236 | 0.930 | 0.454 | 0.287 | 0.418 |
| ***Faecalibacterium*** | 0.462 | 0.231 | -0.168 | 0.238 | 0.105 | 0.308 | 0.088 | 0.622 | 0.287 | 0.392 | 1.000 | 0.413 | 0.294 | 0.510 | 0.608 | 0.455 | 0.566 | 0.545 | 0.294 | 0.119 | 0.119 | -0.205 | 0.420 | 0.140 | 0.224 |
|  | 0.281 | 0.627 | 0.737 | 0.615 | 0.828 | 0.496 | 0.855 | 0.118 | 0.528 | 0.369 | 1.000 | 0.338 | 0.518 | 0.225 | 0.129 | 0.287 | 0.160 | 0.180 | 0.518 | 0.813 | 0.813 | 0.678 | 0.329 | 0.785 | 0.640 |
| ***Fusicatenibacter*** | 0.643 | 0.741 | 0.601 | 0.664 | 0.692 | 0.741 | 0.627 | 0.371 | 0.524 | 0.608 | 0.413 | 1.000 | 0.552 | 0.217 | 0.629 | 0.406 | 0.573 | 0.657 | 0.545 | 0.168 | 0.566 | 0.175 | 0.434 | 0.245 | 0.084 |
|  | 0.113 | **0.050** | 0.136 | 0.097 | 0.077 | **0.050** | 0.118 | 0.404 | 0.209 | 0.129 | 0.338 | 1.000 | 0.175 | 0.653 | 0.118 | 0.351 | 0.159 | 0.103 | 0.180 | 0.737 | 0.160 | 0.727 | 0.316 | 0.602 | 0.855 |
| ***Gemmiger*** | 0.385 | 0.860 | 0.343 | 0.664 | 0.727 | 0.846 | 0.879 | 0.825 | 0.839 | 0.755 | 0.294 | 0.552 | 1.000 | 0.545 | 0.636 | 0.301 | 0.685 | 0.587 | 0.622 | -0.210 | 0.636 | -0.086 | 0.063 | 0.280 | 0.713 |
|  | 0.379 | **0.013** | 0.447 | 0.097 | 0.060 | **0.016** | **0.009** | **0.019** | **0.016** | 0.048 | 0.518 | 0.175 | 1.000 | 0.180 | 0.117 | 0.511 | 0.082 | 0.145 | 0.118 | 0.669 | 0.117 | 0.855 | 0.887 | 0.536 | 0.063 |
| ***Lientehia*** | 0.420 | 0.280 | -0.070 | 0.503 | 0.385 | 0.392 | 0.427 | 0.608 | 0.336 | 0.224 | 0.510 | 0.217 | 0.545 | 1.000 | 0.441 | -0.077 | 0.357 | 0.259 | 0.622 | -0.161 | 0.350 | -0.041 | 0.392 | 0.315 | 0.427 |
|  | 0.329 | 0.536 | 0.876 | 0.234 | 0.379 | 0.369 | 0.327 | 0.129 | 0.454 | 0.640 | 0.225 | 0.653 | 0.180 | 1.000 | 0.306 | 0.867 | 0.432 | 0.576 | 0.118 | 0.744 | 0.440 | 0.924 | 0.369 | 0.488 | 0.327 |
| **NA** | 0.476 | 0.490 | 0.203 | 0.741 | 0.490 | 0.657 | 0.473 | 0.741 | 0.622 | 0.895 | 0.608 | 0.629 | 0.636 | 0.441 | 1.000 | 0.755 | 0.783 | 0.825 | 0.622 | 0.070 | 0.510 | -0.067 | 0.517 | 0.434 | 0.343 |
|  | 0.263 | 0.243 | 0.682 | **0.050** | 0.243 | 0.103 | 0.266 | 0.050 | 0.118 | 0.008 | 0.129 | 0.118 | 0.117 | 0.306 | 1.000 | **0.048** | **0.034** | **0.019** | 0.118 | 0.876 | 0.225 | 0.880 | 0.218 | 0.316 | 0.447 |
| ***Mediterraneibacter*** | 0.189 | 0.245 | -0.098 | 0.448 | 0.105 | 0.287 | 0.119 | 0.545 | 0.371 | 0.692 | 0.455 | 0.406 | 0.301 | -0.077 | 0.755 | 1.000 | 0.678 | 0.832 | 0.168 | 0.056 | 0.252 | -0.243 | 0.147 | 0.161 | 0.266 |
|  | 0.699 | 0.602 | 0.841 | 0.299 | 0.828 | 0.528 | 0.813 | 0.180 | 0.404 | 0.077 | 0.287 | 0.351 | 0.511 | 0.867 | **0.048** | 1.000 | 0.084 | **0.018** | 0.737 | 0.896 | 0.592 | 0.605 | 0.769 | 0.744 | 0.563 |
| ***Parabacteroides*** | 0.524 | 0.594 | 0.175 | 0.566 | 0.350 | 0.608 | 0.497 | 0.678 | 0.762 | 0.720 | 0.566 | 0.573 | 0.685 | 0.357 | 0.783 | 0.678 | 1.000 | 0.720 | 0.462 | 0.273 | 0.552 | 0.082 | 0.343 | 0.350 | 0.503 |
|  | 0.209 | 0.138 | 0.727 | 0.160 | 0.440 | 0.129 | 0.236 | 0.084 | **0.046** | 0.063 | 0.160 | 0.159 | 0.082 | 0.432 | **0.034** | 0.084 | 1.000 | 0.063 | 0.281 | 0.551 | 0.175 | 0.857 | 0.447 | 0.440 | 0.234 |
| ***Phocaeicola*** | 0.483 | 0.497 | -0.021 | 0.650 | 0.329 | 0.483 | 0.354 | 0.692 | 0.420 | 0.790 | 0.545 | 0.657 | 0.587 | 0.259 | 0.825 | 0.832 | 0.720 | 1.000 | 0.497 | -0.154 | 0.322 | -0.138 | 0.343 | 0.336 | 0.280 |
|  | 0.251 | 0.236 | 0.958 | 0.105 | 0.469 | 0.251 | 0.437 | 0.077 | 0.329 | **0.033** | 0.180 | 0.103 | 0.145 | 0.576 | **0.019** | **0.018** | 0.063 | 1.000 | 0.236 | 0.757 | 0.481 | 0.787 | 0.447 | 0.454 | 0.536 |
| ***Romboutsia*** | 0.217 | 0.573 | 0.385 | 0.629 | 0.469 | 0.552 | 0.578 | 0.510 | 0.336 | 0.517 | 0.294 | 0.545 | 0.622 | 0.622 | 0.622 | 0.168 | 0.462 | 0.497 | 1.000 | -0.189 | 0.294 | -0.086 | 0.413 | 0.105 | 0.280 |
|  | 0.653 | 0.159 | 0.379 | 0.118 | 0.273 | 0.175 | 0.157 | 0.225 | 0.454 | 0.218 | 0.518 | 0.180 | 0.118 | 0.118 | 0.118 | 0.737 | 0.281 | 0.236 | 1.000 | 0.699 | 0.518 | 0.855 | 0.338 | 0.828 | 0.536 |
| ***Roseburia*** | 0.315 | -0.175 | 0.196 | -0.056 | -0.147 | -0.084 | -0.042 | -0.308 | 0.112 | -0.021 | 0.119 | 0.168 | -0.210 | -0.161 | 0.070 | 0.056 | 0.273 | -0.154 | -0.189 | 1.000 | 0.154 | 0.295 | 0.315 | 0.322 | -0.266 |
|  | 0.488 | 0.727 | 0.686 | 0.896 | 0.769 | 0.855 | 0.924 | 0.496 | 0.826 | 0.958 | 0.813 | 0.737 | 0.669 | 0.744 | 0.876 | 0.896 | 0.551 | 0.757 | 0.699 | 1.000 | 0.757 | 0.518 | 0.488 | 0.481 | 0.563 |
| ***Ruminococcus*** | 0.483 | 0.629 | 0.594 | 0.818 | 0.839 | 0.783 | 0.715 | 0.441 | 0.818 | 0.497 | 0.119 | 0.566 | 0.636 | 0.350 | 0.510 | 0.252 | 0.552 | 0.322 | 0.294 | 0.154 | 1.000 | 0.246 | 0.196 | 0.378 | 0.566 |
|  | 0.251 | 0.118 | 0.138 | **0.020** | **0.016** | 0.034 | 0.063 | 0.306 | **0.020** | 0.236 | 0.813 | 0.160 | 0.117 | 0.440 | 0.225 | 0.592 | 0.175 | 0.481 | 0.518 | 0.757 | 1.000 | 0.602 | 0.686 | 0.392 | 0.160 |
| ***Segatella*** | 0.593 | -0.015 | 0.317 | 0.060 | 0.131 | 0.116 | -0.110 | -0.422 | 0.127 | -0.037 | -0.205 | 0.175 | -0.086 | -0.041 | -0.067 | -0.243 | 0.082 | -0.138 | -0.086 | 0.295 | 0.246 | 1.000 | 0.593 | 0.537 | -0.440 |
|  | 0.138 | 0.966 | 0.488 | 0.892 | 0.797 | 0.819 | 0.827 | 0.329 | 0.804 | 0.930 | 0.678 | 0.727 | 0.855 | 0.924 | 0.880 | 0.605 | 0.857 | 0.787 | 0.855 | 0.518 | 0.602 | 1.000 | 0.138 | 0.192 | 0.306 |
| ***Streptococcus*** | 0.741 | -0.021 | 0.161 | 0.399 | 0.133 | 0.196 | -0.074 | 0.014 | 0.084 | 0.336 | 0.420 | 0.434 | 0.063 | 0.392 | 0.517 | 0.147 | 0.343 | 0.343 | 0.413 | 0.315 | 0.196 | 0.593 | 1.000 | 0.692 | -0.392 |
|  | **0.050** | 0.958 | 0.744 | 0.362 | 0.794 | 0.686 | 0.873 | 0.966 | 0.855 | 0.454 | 0.329 | 0.316 | 0.887 | 0.369 | 0.218 | 0.769 | 0.447 | 0.447 | 0.338 | 0.488 | 0.686 | 0.138 | 1.000 | 0.077 | 0.369 |
| ***Sutterella*** | 0.776 | -0.028 | -0.014 | 0.455 | 0.196 | 0.196 | 0.109 | 0.126 | 0.287 | 0.455 | 0.140 | 0.245 | 0.280 | 0.315 | 0.434 | 0.161 | 0.350 | 0.336 | 0.105 | 0.322 | 0.378 | 0.537 | 0.692 | 1.000 | -0.133 |
|  | **0.036** | 0.950 | 0.966 | 0.287 | 0.686 | 0.686 | 0.828 | 0.804 | 0.528 | 0.287 | 0.785 | 0.602 | 0.536 | 0.488 | 0.316 | 0.744 | 0.440 | 0.454 | 0.828 | 0.481 | 0.392 | 0.192 | 0.077 | 1.000 | 0.794 |
| ***Vescimonas*** | -0.098 | 0.573 | 0.084 | 0.455 | 0.462 | 0.531 | 0.630 | 0.776 | 0.685 | 0.364 | 0.224 | 0.084 | 0.713 | 0.427 | 0.343 | 0.266 | 0.503 | 0.280 | 0.280 | -0.266 | 0.566 | -0.440 | -0.392 | -0.133 | 1.000 |
|  | 0.841 | 0.159 | 0.855 | 0.287 | 0.281 | 0.200 | 0.118 | **0.036** | 0.082 | 0.418 | 0.640 | 0.855 | 0.063 | 0.327 | 0.447 | 0.563 | 0.234 | 0.536 | 0.536 | 0.563 | 0.160 | 0.306 | 0.369 | 0.794 | 1.000 |

Bacterial genus-genus interactions from the entire cohort. Pairwise correlations between genus using log_2_ (fold change) data were calculated using a Spearman’s rank correlation (two sided adjusted for using BH-FDR). The correlations ranged from -1 to 1 (negative to positive). Red text indicates adjusted *P* (*Q*) values ≤ 0.05 (grey rows).

Genus-genus correlation by group: Maltodextrin

|  | ***Agathobacter*** | ***Anaerobutyricum*** | ***Anaerostipes*** | ***Bacteroides*** | ***Bifidobacterium*** | ***Blautia*** | ***Collinsella*** | ***Coprococcus*** | ***Dialister*** | ***Dorea*** | ***Faecalibacterium*** | ***Fusicatenibacter*** | ***Gemmiger*** | ***Lientehia*** | ***NA*** | ***Mediterraneibacter*** | ***Parabacteroides*** | ***Phocaeicola*** | ***Romboutsia*** | ***Roseburia*** | ***Ruminococcus*** | ***Segatella*** | ***Streptococcus*** | ***Sutterella*** | ***Vescimonas*** |
| --- | --- | --- | --- | --- | --- | --- | --- | --- | --- | --- | --- | --- | --- | --- | --- | --- | --- | --- | --- | --- | --- | --- | --- | --- | --- |
| ***Agathobacter*** | 1.000 | 0.418 | 0.516 | 0.071 | 0.066 | 0.467 | 0.680 | 0.385 | 0.242 | 0.253 | 0.659 | 0.742 | 0.615 | 0.527 | 0.071 | 0.280 | 0.335 | 0.769 | 0.324 | 0.264 | 0.188 | 0.418 | 0.412 | 0.232 | 0.423 |
|  | 1.000 | 0.316 | 0.212 | 0.872 | 0.875 | 0.267 | 0.084 | 0.358 | 0.568 | 0.562 | 0.104 | 0.057 | 0.128 | 0.210 | 0.872 | 0.518 | 0.436 | 0.057 | 0.456 | 0.543 | 0.665 | 0.316 | 0.319 | 0.587 | 0.310 |
| ***Anaerobutyricum*** | 0.418 | 1.000 | 0.242 | 0.269 | 0.181 | 0.747 | 0.304 | 0.286 | 0.440 | 0.423 | 0.560 | 0.220 | 0.610 | 0.110 | 0.489 | 0.478 | 0.648 | 0.253 | 0.478 | 0.121 | 0.227 | 0.753 | 0.319 | 0.657 | 0.522 |
|  | 0.316 | 1.000 | 0.568 | 0.539 | 0.678 | 0.057 | 0.491 | 0.518 | 0.297 | 0.310 | 0.178 | 0.603 | 0.130 | 0.810 | 0.247 | 0.248 | 0.113 | 0.562 | 0.248 | 0.800 | 0.596 | 0.057 | 0.463 | 0.104 | 0.210 |
| ***Anaerostipes*** | 0.516 | 0.242 | 1.000 | 0.516 | 0.434 | 0.484 | 0.895 | 0.621 | 0.568 | 0.742 | 0.181 | 0.775 | 0.451 | 0.731 | 0.621 | 0.560 | 0.462 | 0.725 | -0.154 | -0.313 | -0.011 | 0.137 | 0.813 | 0.066 | 0.209 |
|  | 0.212 | 0.568 | 1.000 | 0.212 | 0.300 | 0.248 | **0.004** | 0.126 | 0.176 | 0.057 | 0.678 | 0.057 | 0.282 | 0.057 | 0.126 | 0.178 | 0.270 | 0.060 | 0.739 | 0.470 | 0.971 | 0.773 | **0.031** | 0.875 | 0.622 |
| ***Bacteroides*** | 0.071 | 0.269 | 0.516 | 1.000 | 0.110 | 0.692 | 0.442 | 0.484 | 0.719 | 0.681 | 0.423 | 0.544 | 0.379 | 0.560 | 0.396 | 0.121 | 0.599 | 0.484 | -0.088 | -0.049 | 0.552 | 0.143 | 0.500 | 0.613 | 0.396 |
|  | 0.872 | 0.539 | 0.212 | 1.000 | 0.810 | 0.077 | 0.297 | 0.248 | 0.063 | 0.084 | 0.310 | 0.191 | 0.366 | 0.178 | 0.339 | 0.800 | 0.143 | 0.248 | 0.840 | 0.893 | 0.188 | 0.759 | 0.232 | 0.129 | 0.339 |
| ***Bifidobacterium*** | 0.066 | 0.181 | 0.434 | 0.110 | 1.000 | 0.253 | 0.282 | 0.225 | 0.279 | 0.286 | -0.209 | 0.082 | -0.038 | -0.104 | 0.703 | 0.313 | 0.363 | -0.060 | 0.110 | -0.418 | -0.122 | 0.143 | 0.456 | -0.110 | 0.099 |
|  | 0.875 | 0.678 | 0.300 | 0.810 | 1.000 | 0.562 | 0.518 | 0.597 | 0.519 | 0.518 | 0.622 | 0.851 | 0.916 | 0.816 | 0.075 | 0.470 | 0.392 | 0.875 | 0.810 | 0.316 | 0.800 | 0.759 | 0.277 | 0.810 | 0.822 |
| ***Blautia*** | 0.467 | 0.747 | 0.484 | 0.692 | 0.253 | 1.000 | 0.481 | 0.297 | 0.638 | 0.615 | 0.736 | 0.527 | 0.742 | 0.467 | 0.489 | 0.280 | 0.775 | 0.516 | 0.302 | 0.220 | 0.431 | 0.735 | 0.407 | 0.757 | 0.478 |
|  | 0.267 | 0.057 | 0.248 | 0.077 | 0.562 | 1.000 | 0.248 | 0.500 | 0.120 | 0.128 | 0.057 | 0.210 | 0.057 | 0.267 | 0.247 | 0.518 | 0.057 | 0.212 | 0.493 | 0.603 | 0.301 | 0.057 | 0.325 | 0.057 | 0.248 |
| ***Collinsella*** | 0.680 | 0.304 | 0.895 | 0.442 | 0.282 | 0.481 | 1.000 | 0.635 | 0.524 | 0.669 | 0.337 | 0.829 | 0.541 | 0.718 | 0.453 | 0.459 | 0.431 | 0.762 | 0.061 | -0.298 | 0.267 | 0.120 | 0.702 | 0.061 | 0.464 |
|  | 0.084 | 0.491 | **0.004** | 0.297 | 0.518 | 0.248 | 1.000 | 0.120 | 0.210 | 0.096 | 0.436 | **0.023** | 0.193 | 0.063 | 0.281 | 0.274 | 0.301 | 0.057 | 0.875 | 0.500 | 0.543 | 0.800 | 0.075 | 0.875 | 0.270 |
| ***Coprococcus*** | 0.385 | 0.286 | 0.621 | 0.484 | 0.225 | 0.297 | 0.635 | 1.000 | 0.635 | 0.593 | 0.335 | 0.736 | 0.385 | 0.593 | 0.390 | 0.505 | 0.363 | 0.560 | 0.280 | -0.231 | 0.331 | -0.203 | 0.841 | 0.177 | 0.659 |
|  | 0.358 | 0.518 | 0.126 | 0.248 | 0.597 | 0.500 | 0.120 | 1.000 | 0.120 | 0.146 | 0.436 | 0.057 | 0.358 | 0.146 | 0.350 | 0.230 | 0.392 | 0.178 | 0.518 | 0.587 | 0.443 | 0.635 | **0.019** | 0.687 | 0.104 |
| ***Dialister*** | 0.242 | 0.440 | 0.568 | 0.719 | 0.279 | 0.638 | 0.524 | 0.635 | 1.000 | 0.513 | 0.446 | 0.655 | 0.359 | 0.627 | 0.733 | 0.618 | 0.524 | 0.435 | 0.134 | -0.176 | 0.342 | 0.242 | 0.549 | 0.574 | 0.685 |
|  | 0.568 | 0.297 | 0.176 | 0.063 | 0.519 | 0.120 | 0.210 | 0.120 | 1.000 | 0.218 | 0.291 | 0.106 | 0.397 | 0.126 | 0.057 | 0.128 | 0.210 | 0.300 | 0.780 | 0.688 | 0.429 | 0.568 | 0.191 | 0.170 | 0.083 |
| ***Dorea*** | 0.253 | 0.423 | 0.742 | 0.681 | 0.286 | 0.615 | 0.669 | 0.593 | 0.513 | 1.000 | 0.280 | 0.544 | 0.500 | 0.522 | 0.522 | 0.264 | 0.495 | 0.434 | -0.066 | -0.049 | 0.282 | 0.251 | 0.593 | 0.320 | 0.297 |
|  | 0.562 | 0.310 | 0.057 | 0.084 | 0.518 | 0.128 | 0.096 | 0.146 | 0.218 | 1.000 | 0.518 | 0.191 | 0.232 | 0.210 | 0.210 | 0.543 | 0.241 | 0.300 | 0.875 | 0.893 | 0.518 | 0.562 | 0.146 | 0.463 | 0.500 |
| ***Faecalibacterium*** | 0.659 | 0.560 | 0.181 | 0.423 | -0.209 | 0.736 | 0.337 | 0.335 | 0.446 | 0.280 | 1.000 | 0.610 | 0.764 | 0.500 | -0.016 | 0.104 | 0.396 | 0.588 | 0.571 | 0.566 | 0.575 | 0.544 | 0.214 | 0.740 | 0.621 |
|  | 0.104 | 0.178 | 0.678 | 0.310 | 0.622 | 0.057 | 0.436 | 0.436 | 0.291 | 0.518 | 1.000 | 0.130 | 0.057 | 0.232 | 0.961 | 0.816 | 0.339 | 0.150 | 0.172 | 0.178 | 0.170 | 0.191 | 0.615 | 0.057 | 0.126 |
| ***Fusicatenibacter*** | 0.742 | 0.220 | 0.775 | 0.544 | 0.082 | 0.527 | 0.829 | 0.736 | 0.655 | 0.544 | 0.610 | 1.000 | 0.626 | 0.912 | 0.280 | 0.429 | 0.434 | 0.901 | 0.148 | 0.044 | 0.343 | 0.102 | 0.698 | 0.265 | 0.544 |
|  | 0.057 | 0.603 | 0.057 | 0.191 | 0.851 | 0.210 | 0.023 | 0.057 | 0.106 | 0.191 | 0.130 | 1.000 | 0.126 | **0.004** | 0.518 | 0.304 | 0.300 | **0.004** | 0.751 | 0.905 | 0.429 | 0.821 | 0.075 | 0.543 | 0.191 |
| ***Gemmiger*** | 0.615 | 0.610 | 0.451 | 0.379 | -0.038 | 0.742 | 0.541 | 0.385 | 0.359 | 0.500 | 0.764 | 0.626 | 1.000 | 0.637 | 0.132 | 0.374 | 0.643 | 0.698 | 0.538 | 0.363 | 0.414 | 0.544 | 0.451 | 0.398 | 0.412 |
|  | 0.128 | 0.130 | 0.282 | 0.366 | 0.916 | 0.057 | 0.193 | 0.358 | 0.397 | 0.232 | 0.057 | 0.126 | 1.000 | 0.120 | 0.781 | 0.377 | 0.119 | 0.075 | 0.196 | 0.392 | 0.318 | 0.191 | 0.282 | 0.339 | 0.319 |
| ***Lientehia*** | 0.527 | 0.110 | 0.731 | 0.560 | -0.104 | 0.467 | 0.718 | 0.593 | 0.627 | 0.522 | 0.500 | 0.912 | 0.637 | 1.000 | 0.236 | 0.462 | 0.401 | 0.857 | -0.022 | 0.060 | 0.221 | 0.072 | 0.588 | 0.199 | 0.341 |
|  | 0.210 | 0.810 | 0.057 | 0.178 | 0.816 | 0.267 | 0.063 | 0.146 | 0.126 | 0.210 | 0.232 | 0.004 | 0.120 | 1.000 | 0.580 | 0.270 | 0.335 | **0.014** | 0.953 | 0.875 | 0.603 | 0.872 | 0.150 | 0.643 | 0.429 |
| **NA** | 0.071 | 0.489 | 0.621 | 0.396 | 0.703 | 0.489 | 0.453 | 0.390 | 0.733 | 0.522 | -0.016 | 0.280 | 0.132 | 0.236 | 1.000 | 0.714 | 0.462 | 0.104 | -0.016 | -0.440 | -0.110 | 0.352 | 0.522 | 0.271 | 0.319 |
|  | 0.872 | 0.247 | 0.126 | 0.339 | 0.075 | 0.247 | 0.281 | 0.350 | 0.057 | 0.210 | 0.961 | 0.518 | 0.781 | 0.580 | 1.000 | 0.065 | 0.270 | 0.816 | 0.961 | 0.297 | 0.810 | 0.407 | 0.210 | 0.538 | 0.463 |
| ***Mediterraneibacter*** | 0.280 | 0.478 | 0.560 | 0.121 | 0.313 | 0.280 | 0.459 | 0.505 | 0.618 | 0.264 | 0.104 | 0.429 | 0.374 | 0.462 | 0.714 | 1.000 | 0.407 | 0.379 | 0.209 | -0.286 | -0.193 | 0.251 | 0.604 | 0.110 | 0.330 |
|  | 0.518 | 0.248 | 0.178 | 0.800 | 0.470 | 0.518 | 0.274 | 0.230 | 0.128 | 0.543 | 0.816 | 0.304 | 0.377 | 0.270 | 0.065 | 1.000 | 0.325 | 0.366 | 0.622 | 0.518 | 0.653 | 0.562 | 0.137 | 0.810 | 0.445 |
| ***Parabacteroides*** | 0.335 | 0.648 | 0.462 | 0.599 | 0.363 | 0.775 | 0.431 | 0.363 | 0.524 | 0.495 | 0.396 | 0.434 | 0.643 | 0.401 | 0.462 | 0.407 | 1.000 | 0.516 | 0.231 | -0.033 | 0.354 | 0.484 | 0.555 | 0.414 | 0.363 |
|  | 0.436 | 0.113 | 0.270 | 0.143 | 0.392 | 0.057 | 0.301 | 0.392 | 0.210 | 0.241 | 0.339 | 0.300 | 0.119 | 0.335 | 0.270 | 0.325 | 1.000 | 0.212 | 0.587 | 0.927 | 0.407 | 0.248 | 0.186 | 0.318 | 0.392 |
| ***Phocaeicola*** | 0.769 | 0.253 | 0.725 | 0.484 | -0.060 | 0.516 | 0.762 | 0.560 | 0.435 | 0.434 | 0.588 | 0.901 | 0.698 | 0.857 | 0.104 | 0.379 | 0.516 | 1.000 | 0.088 | 0.093 | 0.249 | 0.173 | 0.681 | 0.243 | 0.313 |
|  | 0.057 | 0.562 | 0.060 | 0.248 | 0.875 | 0.212 | 0.057 | 0.178 | 0.300 | 0.300 | 0.150 | 0.004 | 0.075 | 0.014 | 0.816 | 0.366 | 0.212 | 1.000 | 0.840 | 0.834 | 0.563 | 0.691 | 0.084 | 0.568 | 0.470 |
| ***Romboutsia*** | 0.324 | 0.478 | -0.154 | -0.088 | 0.110 | 0.302 | 0.061 | 0.280 | 0.134 | -0.066 | 0.571 | 0.148 | 0.538 | -0.022 | -0.016 | 0.209 | 0.231 | 0.088 | 1.000 | 0.247 | 0.503 | 0.257 | 0.154 | 0.249 | 0.621 |
|  | 0.456 | 0.248 | 0.739 | 0.840 | 0.810 | 0.493 | 0.875 | 0.518 | 0.780 | 0.875 | 0.172 | 0.751 | 0.196 | 0.953 | 0.961 | 0.622 | 0.587 | 0.840 | 1.000 | 0.564 | 0.232 | 0.559 | 0.739 | 0.563 | 0.126 |
| ***Roseburia*** | 0.264 | 0.121 | -0.313 | -0.049 | -0.418 | 0.220 | -0.298 | -0.231 | -0.176 | -0.049 | 0.566 | 0.044 | 0.363 | 0.060 | -0.440 | -0.286 | -0.033 | 0.093 | 0.247 | 1.000 | 0.193 | 0.484 | -0.368 | 0.409 | -0.060 |
|  | 0.543 | 0.800 | 0.470 | 0.893 | 0.316 | 0.603 | 0.500 | 0.587 | 0.688 | 0.893 | 0.178 | 0.905 | 0.392 | 0.875 | 0.297 | 0.518 | 0.927 | 0.834 | 0.564 | 1.000 | 0.653 | 0.248 | 0.388 | 0.324 | 0.875 |
| ***Ruminococcus*** | 0.188 | 0.227 | -0.011 | 0.552 | -0.122 | 0.431 | 0.267 | 0.331 | 0.342 | 0.282 | 0.575 | 0.343 | 0.414 | 0.221 | -0.110 | -0.193 | 0.354 | 0.249 | 0.503 | 0.193 | 1.000 | 0.054 | 0.088 | 0.433 | 0.696 |
|  | 0.665 | 0.596 | 0.971 | 0.188 | 0.800 | 0.301 | 0.543 | 0.443 | 0.429 | 0.518 | 0.170 | 0.429 | 0.318 | 0.603 | 0.810 | 0.653 | 0.407 | 0.563 | 0.232 | 0.653 | 1.000 | 0.887 | 0.840 | 0.300 | 0.075 |
| ***Segatella*** | 0.418 | 0.753 | 0.137 | 0.143 | 0.143 | 0.735 | 0.120 | -0.203 | 0.242 | 0.251 | 0.544 | 0.102 | 0.544 | 0.072 | 0.352 | 0.251 | 0.484 | 0.173 | 0.257 | 0.484 | 0.054 | 1.000 | -0.060 | 0.631 | 0.131 |
|  | 0.316 | 0.057 | 0.773 | 0.759 | 0.759 | 0.057 | 0.800 | 0.635 | 0.568 | 0.562 | 0.191 | 0.821 | 0.191 | 0.872 | 0.407 | 0.562 | 0.248 | 0.691 | 0.559 | 0.248 | 0.887 | 1.000 | 0.875 | 0.125 | 0.781 |
| ***Streptococcus*** | 0.412 | 0.319 | 0.813 | 0.500 | 0.456 | 0.407 | 0.702 | 0.841 | 0.549 | 0.593 | 0.214 | 0.698 | 0.451 | 0.588 | 0.522 | 0.604 | 0.555 | 0.681 | 0.154 | -0.368 | 0.088 | -0.060 | 1.000 | 0.099 | 0.357 |
|  | 0.319 | 0.463 | **0.031** | 0.232 | 0.277 | 0.325 | 0.075 | **0.019** | 0.191 | 0.146 | 0.615 | 0.075 | 0.282 | 0.150 | 0.210 | 0.137 | 0.186 | 0.084 | 0.739 | 0.388 | 0.840 | 0.875 | 1.000 | 0.822 | 0.400 |
| ***Sutterella*** | 0.232 | 0.657 | 0.066 | 0.613 | -0.110 | 0.757 | 0.061 | 0.177 | 0.574 | 0.320 | 0.740 | 0.265 | 0.398 | 0.199 | 0.271 | 0.110 | 0.414 | 0.243 | 0.249 | 0.409 | 0.433 | 0.631 | 0.099 | 1.000 | 0.481 |
|  | 0.587 | 0.104 | 0.875 | 0.129 | 0.810 | 0.057 | 0.875 | 0.687 | 0.170 | 0.463 | 0.057 | 0.543 | 0.339 | 0.643 | 0.538 | 0.810 | 0.318 | 0.568 | 0.563 | 0.324 | 0.300 | 0.125 | 0.822 | 1.000 | 0.248 |
| ***Vescimonas*** | 0.423 | 0.522 | 0.209 | 0.396 | 0.099 | 0.478 | 0.464 | 0.659 | 0.685 | 0.297 | 0.621 | 0.544 | 0.412 | 0.341 | 0.319 | 0.330 | 0.363 | 0.313 | 0.621 | -0.060 | 0.696 | 0.131 | 0.357 | 0.481 | 1.000 |
|  | 0.310 | 0.210 | 0.622 | 0.339 | 0.822 | 0.248 | 0.270 | 0.104 | 0.083 | 0.500 | 0.126 | 0.191 | 0.319 | 0.429 | 0.463 | 0.445 | 0.392 | 0.470 | 0.126 | 0.875 | 0.075 | 0.781 | 0.400 | 0.248 | 1.000 |

Bacterial genus-genus interactions from the entire cohort. Pairwise correlations between genus using log_2_ (fold change) data were calculated using a Spearman’s rank correlation (two sided adjusted for using BH-FDR). The correlations ranged from -1 to 1 (negative to positive). Red text indicates adjusted *P* (*Q*) values ≤ 0.05 (grey rows).

## **Supplementary Table 5 Mean organic acid concentration across all interventions at V1 and V5 of the intervention phase**

|  |  | | | **Intervention** | | |  | | |
| --- | --- | --- | --- | --- | --- | --- | --- | --- | --- |
| **Organic acid** | **Cellobiose (n = 12)** | | | **OF P95 (n = 12)** | | | **Maltodextrin ( n = 13)** | | |
|  | **V1** | **V5** | ***P*** | **V1** | **V5** | ***P*** | **V1** | **V5** | ***P*** |
| **Acetate** | 20.459  (3.035) | 21.032  (2.672) | 0.833 | 25.527  (3.035) | 19.559  (2.672) | **0.033** | 21.908  (2.916) | 18.794  (2.567) | 0.236 |
| **Butyrate** | 5.955  (1.265) | 6.425  (1.213) | 0.698 | 8.163  (1.265) | 5.664  (1.213) | **0.045** | 7.033  (1.215) | 6.874  (1.116) | 0.891 |
| **Propionate** | 7.473  (1.064) | 8.032  (1.258) | 0.541 | 7.701  (1.064) | 6.411  (1.258) | 0.164 | 7.304  (1.023) | 5.895  (1.209) | 0.115 |
| **Lactate** | 0 | 0 | 1.000 | 0 | 0 | 1.000 | 0 | 0 | 1.000 |
| **Isovalerate** | 0 | 0 | 1.000 | 0.137  (0.124) | 0.217  (0.124) | 0.657 | 0.050  (0.125) | 0.097  (0.147) | 0.173 |
| **Valerate** | 0.130  (0.203) | 0.259  (0.288) | 0.611 | 0.611  (0.203) | 0.535 | 0.535 | 0.227  (0.195) | 0.438  (0.277) | 0.387 |
| **Isobutytate** | 0 | 0 | 1.000 | 0.027  (0.099) | 0 | 0.234 | 0.385  (0.111) | 0.093  (0.174) | 0.518 |
| **Total organic acid** | 34.018  (4.791) | 35.749  (4.959) | 0.689 | 42.283  (4.791) | 33.003  (4.959) | **0.038** | 36.826  (4.603) | 32.001  (4.765) | 0.250 |

## **Supplementary Table 6 Bacterial genus-SCFA correlation matrix (overall)**

|  | ***Agathobacter*** | ***Anaerobutyricum*** | ***Anaerostipes*** | ***Bacteroides*** | ***Bifidobacterium*** | ***Blautia*** | ***Collinsella*** | ***Coprococcus*** | ***Dialister*** | ***Dorea*** | ***Faecalibacterium*** | ***Fusicatenibacter*** | ***Gemmiger*** | ***Lientehia*** | ***NA*** | ***Mediterraneibacter*** | ***Parabacteroides*** | ***Phocaeicola*** | ***Romboutsia*** | ***Roseburia*** | ***Ruminococcus*** | ***Segatella*** | ***Streptococcus*** | ***Sutterella*** | ***Vescimonas*** |
| --- | --- | --- | --- | --- | --- | --- | --- | --- | --- | --- | --- | --- | --- | --- | --- | --- | --- | --- | --- | --- | --- | --- | --- | --- | --- |
| **Acetate** | 0.074 | 0.113 | 0.103 | -0.029 | 0.266 | 0.033 | 0.259 | 0.049 | -0.196 | 0.090 | 0.240 | 0.174 | 0.143 | 0.198 | 0.023 | 0.144 | 0.033 | 0.094 | 0.309 | 0.337 | 0.006 | 0.227 | 0.147 | 0.070 | -0.079 |
|  | 0.978 | 0.978 | 0.978 | 0.979 | 0.916 | 0.979 | 0.916 | 0.979 | 0.916 | 0.978 | 0.916 | 0.968 | 0.978 | 0.916 | 0.979 | 0.978 | 0.979 | 0.978 | 0.916 | 0.916 | 0.994 | 0.916 | 0.978 | 0.978 | 0.978 |
| **Butyrate** | -0.136 | 0.189 | 0.179 | -0.134 | 0.221 | 0.057 | 0.011 | -0.115 | -0.223 | -0.001 | 0.050 | 0.264 | -0.001 | -0.019 | 0.119 | -0.098 | -0.191 | 0.100 | 0.158 | 0.194 | -0.069 | -0.086 | 0.077 | -0.216 | -0.121 |
|  | 0.978 | 0.916 | 0.964 | 0.978 | 0.916 | 0.979 | 0.988 | 0.978 | 0.916 | 0.994 | 0.979 | 0.916 | 0.994 | 0.979 | 0.978 | 0.978 | 0.916 | 0.978 | 0.978 | 0.916 | 0.978 | 0.978 | 0.978 | 0.916 | 0.978 |
| **Propionate** | 0.015 | -0.221 | -0.058 | -0.266 | -0.300 | -0.147 | -0.188 | -0.097 | -0.108 | -0.141 | 0.118 | -0.024 | -0.100 | -0.087 | -0.040 | -0.204 | -0.203 | -0.160 | -0.275 | 0.136 | -0.003 | 0.041 | 0.080 | 0.060 | -0.227 |
|  | 0.985 | 0.916 | 0.979 | 0.916 | 0.916 | 0.978 | 0.916 | 0.978 | 0.978 | 0.978 | 0.978 | 0.979 | 0.978 | 0.978 | 0.979 | 0.916 | 0.916 | 0.978 | 0.916 | 0.978 | 0.994 | 0.979 | 0.978 | 0.979 | 0.916 |
| **Total SCFA** | -0.030 | -0.014 | 0.082 | -0.050 | 0.170 | 0.021 | 0.043 | -0.023 | -0.238 | -0.105 | 0.168 | 0.202 | 0.029 | 0.259 | 0.072 | 0.047 | -0.051 | 0.043 | 0.110 | 0.237 | -0.095 | 0.083 | 0.226 | 0.047 | -0.188 |
|  | 0.979 | 0.985 | 0.978 | 0.979 | 0.968 | 0.979 | 0.979 | 0.979 | 0.916 | 0.978 | 0.968 | 0.916 | 0.979 | 0.916 | 0.978 | 0.979 | 0.979 | 0.979 | 0.978 | 0.916 | 0.978 | 0.978 | 0.916 | 0.979 | 0.916 |

Bacterial genus-SCFA correlation from the entire cohort. Pairwise correlations between genus using log_2_ (fold change) data were calculated using a Spearman’s rank correlation (two-sided adjusted for using BH-FDR). The correlations ranged from -1 to 1 (negative to positive). Grey rows indicated adjusted *P* (*Q*) values.
